# Legionella pneumophila PPIase Mip Interacts with the Bacterial Proteins SspB, Lpc2061, and FlaA and Promotes Flagellation

**DOI:** 10.1128/iai.00276-22

**Published:** 2022-10-31

**Authors:** Mustafa Safa Karagöz, Can Murat Ünal, Benjamin E. Mayer, Mathias Müsken, José Manuel Borrero-de Acuña, Michael Steinert

**Affiliations:** a Institut für Mikrobiologie, Technische Universität Braunschweig, Braunschweig, Germany; b Computational Biology and Simulation, Technische Universität Darmstadt, Darmstadt, Germany; c Helmholtz Centre for Infection Research, Braunschweig, Germany; d Departamento de Microbiología, Facultad de Biología, Universidad de Seville, Seville, Spain; Yale University School of Medicine

**Keywords:** *Legionella pneumophila*, macrophage infectivity potentiator (Mip), peptidyl-prolyl *cis*/*trans* isomerase (PPIase), flagella, interactomics

## Abstract

The peptidyl-prolyl-*cis/trans*-isomerase (PPIase) macrophage infectivity potentiator (Mip) contributes to the pathogenicity and fitness of L. pneumophila, the causative agent of Legionnaires’ disease. Here, we identified the stringent starvation protein SspB, hypothetical protein Lpc2061, and flagellin FlaA as bacterial interaction partners of Mip. The macrolide FK506, which inhibits the PPIase activity of Mip, interfered with the binding of Lpc2061. Moreover, we demonstrated that the N-terminal dimerization region and amino acid Y185 in the C-terminal PPIase domain of Mip are required for the binding of Lpc2061 and FlaA. The modeling of the interaction partners and global docking with Mip suggested nonoverlapping binding interfaces, and a molecular dynamic simulation predicted an increased stability for the tripartite interaction of Lpc2061, Mip, and FlaA. On the functional level, we demonstrated that Mip promotes L. pneumophila flagellation, which is positively influenced by the binding of Lpc2061 and reduced by FK506. Also, L. pneumophila mutants expressing the Y185A or the monomeric Mip variant, which bind less Lpc2061, were nonmotile, were less flagellated, and yielded less FlaA when quantified. To our knowledge, this is the first report in which a PPIase and its bacterial interaction partners were demonstrated to influence flagellation.

## INTRODUCTION

L. pneumophila, the causative agent of Legionnaires’ disease, naturally inhabits freshwaters and accumulates in biofilms, where it parasitizes intracellularly within different protozoa species (1–3). L. pneumophila also thrives in human-made water systems, including air conditioning units and cooling towers (4–7). Infection of humans occurs via the inhalation of L. pneumophila-contaminated aerosols, which can lead to severe and life-threatening pneumonia. Upon inhalation, the bacteria mainly invade alveolar macrophages, replicate within a specialized cellular compartment, termed the *Legionella*-containing vacuole (LCV), and cause severe lung damage (8–11).

L. pneumophila expresses the macrophage infectivity potentiator (Mip) protein, which was the first genetically identified virulence factor of L. pneumophila (12, 13). Deletion of *mip* results in reduced intracellular replication rates in human alveolar macrophages and protozoa during the onset of infection (12, 14, 15). Mip is a basic 24 kDa surface protein (pI 9.8) and possesses an N-terminal signal sequence that is cleaved off while the protein is transported through the cytoplasmic membrane (16). The protein forms a stable homodimer, and the 2.4 Å crystal structure revealed that each monomer consists of an N-terminal dimerization module, a long connecting α-helix (α3), and a C-terminal peptidyl-prolyl-*cis/trans*-isomerase (PPIase) domain (17–20). The fold of the C-terminal domain (residues 100 to 213) is closely related to the human FK506-binding protein 12 (FKBP12) (21, 22). As characteristic of this protein family, the PPIase activity of Mip is inhibited by the macrolides FK506 and rapamycin (23). In a previous nuclear magnetic resonance (NMR) investigation, we were able to solve the solution structure of free Mip^77-213^ and the Mip^77-213^/rapamycin complex, confirming their stable interaction (24). Mediated by a hinge in the long α-helix, both FKBP domains of the dimerized Mip are subject to large fluctuating movements, which allows for the flexible cooperative binding of potential target structures (25).

Previous studies employing genetic and biochemical methods together with different infection models revealed that Mip impacts the course and outcome of infection on multiple levels (16, 19, 26, 27). Since the PPIase domains of all PPIases contain highly conserved amino acids within their FK506-binding pockets, we generated single substitution mutants, in which Asp142 was replaced by leucine and Tyr185 was replaced by alanine. The purified recombinant proteins exhibited a pronounced loss of PPIase activity in *in vitro* PPIase assays (6.2% for the D142L mutant and 2.0% for the Y185A mutant). When the same site-specifically mutated variants of *mip* were used to complement the L. pneumophila Δ*mip* mutants in infection studies, wild-type phenotypes with Acanthamoeba castellanii or human macrophage-like cell lines were observed (16). We concluded that either additional properties other than the PPIase activity are important during intracellular infection or the residual enzymatic activity of the mutated Mip was still sufficient for the PPIase-dependent phenotypes. In contrast, L. pneumophila strains that were unable to dimerize or had a low PPIase activity were significantly attenuated in a guinea pig infection model (19). This was further confirmed when Mip-deficient bacteria were found to be attenuated and unable to disseminate systemically in guinea pigs (26).

The different effects of a reduction in PPIase activity in cell culture systems and guinea pig infections suggested additional functions of Mip during the more complex infection of guinea pigs. In accordance, the apicobasal transmigration of L. pneumophila *Δmip* mutants were strongly impaired in *in vitro* assays that modeled the lung epithelial barrier, including the extracellular matrix (ECM). Further systematic biochemical binding studies revealed that Mip binds to collagen IV in the ECM. This suggests that there is a concerted action of Mip and proteolytic enzymes that leads to ECM-degradation and transmigration (26). The Mip-collagen IV interaction was further elucidated by utilizing peptide arrays and coprecipitation studies, and a distinct 13 amino acid-long sequence (IPPCPSGWSSLWI; P290) was identified within the globular non-collagenous (NC1) region of collagen IV as the target of Mip. Synthetic P290 interfered with Mip-binding to collagen IV in a dose-dependent manner and accordingly reduced *in vitro* bacterial transmigration. NMR studies of the P290-Mip complex revealed that with its central residues, P290, like rapamycin, occupies the catalytic cleft, thereby forming a hairpin. Its terminal residues make contact with the amino acids of Mip that are outside the catalytic groove, which further stabilizes the complex (28).

Although being the first identified virulence factor of L. pneumophila and the first virulence-associated PPIase, it is still not known how Mip exerts its diverse functions. In this study, we use an interactomic approach and show that stringent starvation protein B (SspB, LPC_0434), hypothetical protein Lpc2061 (LPC_2061), and flagellin (FlaA, LPC_0756) are *in vivo* bacterial interaction partners of Mip. After the validation of these L. pneumophila proteins, we demonstrate that FK506 inhibits the Mip-Lpc2061 interaction. We also report that the dimerization region and the amino acid Y185 of Mip are crucial for the binding of Lpc2061, which is strengthened by FlaA. Via the modeling of the respective interaction partners with AlphaFold v2.0 and the global *in silico* docking of each partner with Mip, we further portray the putative binding interfaces. We demonstrate that Mip-deficient bacterial cells yield less FlaA protein and result in reduced flagellum formation. This is, to our knowledge, the first report showing the promoting effect of a PPIase on bacterial flagellation.

## RESULTS

### Immunoprecipitation reveals stringent starvation protein SspB, hypothetical protein Lpc2061, and flagellin (FlaA) as *in vivo* interaction partners of Mip.

**FIG 1:**
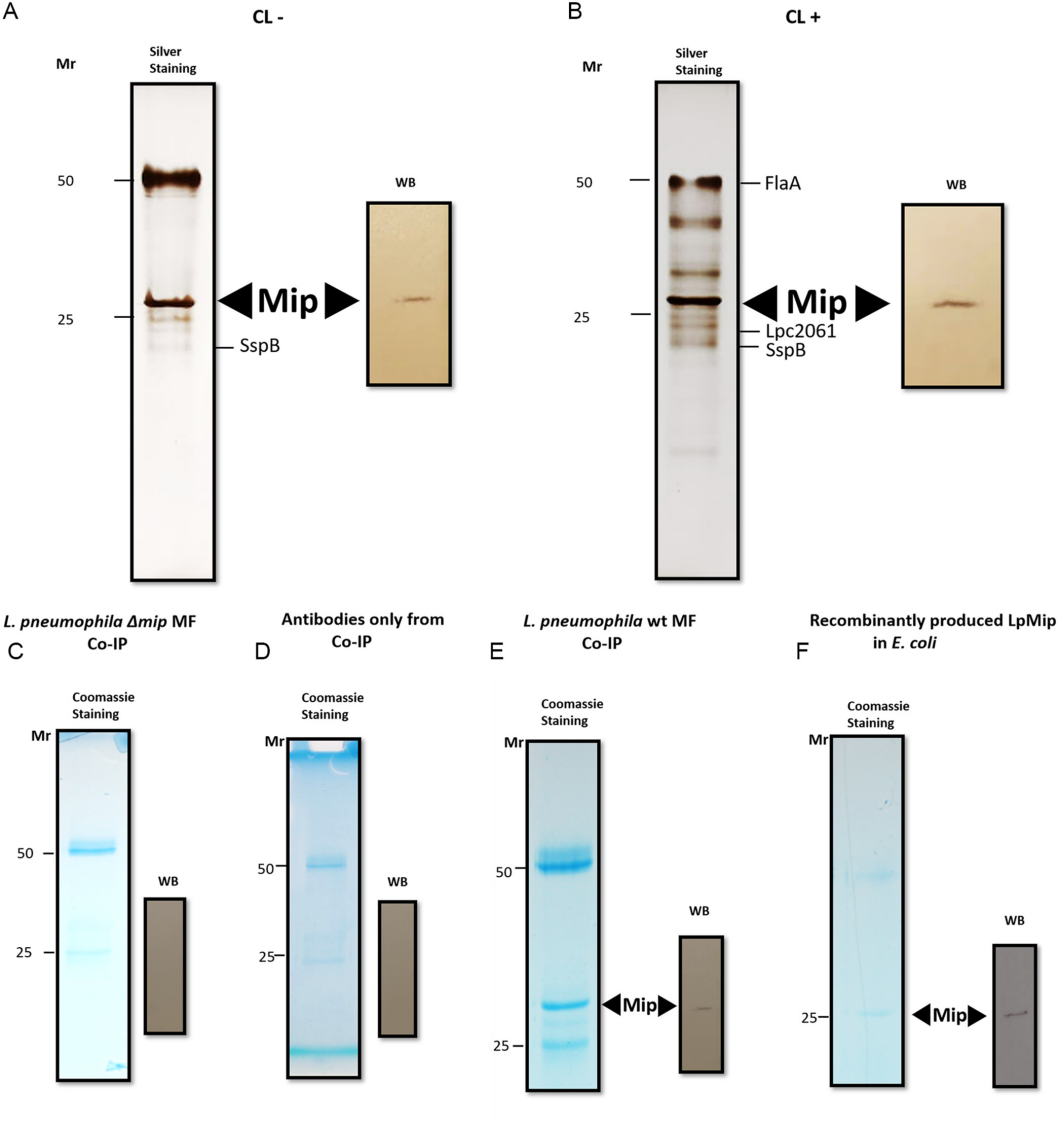
Identification of bacterial interaction partners of L. pneumophila Mip by coimmunoprecipitation. Bacterial cell membrane fractions (MF) were solubilized and coimmunoprecipitated with a mixture of monoclonal Mip-antibodies 2D8 and 22/1. Eluted proteins were resolved by SDS-PAGE and silver stained. (A) Mip eluates from not cross-linked (CL−) homogenates of L. pneumophila cells that were grown to the prestationary-phase resulted in a band at 28 kDa, which corresponds to Mip (left). The identity of Mip was confirmed via the Western blotting (WB) of the same fraction with anti-Mip antibodies (right). (B) To identify additional weaker interaction partners of Mip *in vivo*, cross-linking (CL+) with 0.5% (vol/vol) formaldehyde was performed prior to coimmunoprecipitation, SDS-PAGE, silver staining (left), and Western blotting (right). The CL gave rise to additional protein bands. The purified bands from the wild-type bacteria with and without formaldehyde cross-linking were subsequently subjected to LC/MS analyses for protein identification. Coomassie stained SDS gels and Western blots after coimmunoprecipitation (CoIP) revealed a 25 kDa band, which refers to purified recombinant Mip (C–F). Moreover, the elution fractions showed heavy and light chains of antibodies at 50 and 25 kDa in the Coomassie stains. For the uncropped Western blots, see Fig. S1.

To identify *in vivo* interaction partners of Mip, a mixture of two monoclonal Mip-antibodies, 2D8 and 22/1, was used for coimmunoprecipitation. While antibody 2D8 binds specifically to a C-terminal epitope, antibody 22/1 recognizes an epitope at the N terminus of Mip (unpublished data). Bacterial cell membranes of the wild-type strain L. pneumophila Corby and the corresponding isogenic Δ*mip* mutant strain were solubilized and coimmunoprecipitated (Table 1). Eluted proteins were resolved by SDS-PAGE and stained with silver or Coomassie blue (Fig. 1A–F). As controls, antibodies without any cell material and recombinant Mip were visualized on the gel. Bands at 50 and 25 kDa originated from the light and heavy chains of the antibodies, respectively, and were excluded from the further assessment of interaction partners. Protein eluates derived from membrane fractions of the L. pneumophila wild-type strain showed a clear band at a relative molecular weight of 28 kDa (Fig. 1A), where the recombinantly produced Mip (25 kDa) was detected as well, though this was absent in the Δ*mip* strain (Fig. S1). Additionally, we expanded the identification of the Mip-specific interactome by implementing *in vivo* formaldehyde (FA)-driven cross-linking. This cross-linking should identify interaction partners which bind to a lower extent, with weaker strength, where the interaction is transient, or where partners are simply pulled away by stringent washing conditions. Thus, cultures were alternatively treated with different concentrations of FA as a cross-linking agent prior to cell harvesting and fractionation, followed by the detergent-solubilization of the membrane fractions. Cross-linking with different FA concentrations gave rise to additional protein bands, suggesting further interaction partners. Cross-linking with 0.5% (vol/vol) FA revealed the largest number of protein bands (Fig. 1B), many of which vanished progressively with higher concentrations (Fig. S2). Accordingly, immunodetection assays employing wild-type-derived membrane fractions revealed the major chromogenic signal at a relative molecular weight of 28 kDa, which corresponds to Mip and indicates proper purification (Fig. 1B). Additionally, the L. pneumophila *Δmip* mutant produced neither Mip nor additional bands when superimposed upon the stand-alone antibody fraction, as visualized by silver-staining. This suggests that no unspecific binding of proteins to the column material occurred (Fig. S1 and S2). Coomassie stained SDS gels and Western blots (WB) after coimmunoprecipitation (CoIP) also revealed a 25 kDa band, which refers to purified recombinant Mip (Fig. 1C–F).

**TABLE 1:** Strains and primers used in this study

Strain	Specification	Reference
Legionella pneumophila Corby wt	Media without antibiotics	74
Legionella pneumophila Corby *Δmip*	Kanamycin resistant	16
Legionella pneumophila Corby *ΔflaA*	Kanamycin resistant	55
L. pneumophila Philadelphia 1 JR32-2.3 (Mip D142L)	Kanamycin, chloramphenicol, and streptomycin resistant	16
L. pneumophila Philadelphia 1 JR32-2.2 (Mip Y185A)	Kanamycin, chloramphenicol, and streptomycin resistant	16
L. pneumophila Philadelphia 1 JR32-2.4 (Mip monomer)	Kanamycin, chloramphenicol, and streptomycin resistant	19
E. coli DH10β		
E. coli BL21		
pet52b with LpSspB in E. coli BL21	Ampicilin resistant for the expression of LpSspB with StrepTag	This study
pet22b with Lpc_2061 in E. coli BL21	Ampicilin resistant for the expression Lpc2061 with HisTag	This study
Primers		
AGCGTCGACATGGCAATGACATCAAACAAACC	sspb_fw_SalI	
AGCGAGCTCCTACTTTACCAGTTTTAGTGATGG	sspb_rev_SacI	
AGCGAGCTCAGCCCTGTAGGTCTGATTGTA	2061_nosig_SacI_fv	
AGCGTCGACTTAACTTTTTAGACCAACTGGGAAAA	2061_rv_SalI	

To identify the Mip interaction partners, all of the eluted proteins from wild-type bacteria, including those with and without FA supplementation, were subjected to LC/MS (Table 2). SspB (LPC_0434), a 14.6 kDa protein according to MS, coeluted with Mip without adding cross-linking agents, as determined by LC-MS/MS analysis. Cross-linking experiments coupled with LC-MS/MS analysis led to the identification of hypothetical protein Lpc2061 at 15.7 kDa and of the main flagellar monomer FlaA (LPC_0756) at approximately 48 kDa. SspB (LPC_0434) also coeluted after cross-linking. Interestingly, the silver-stained gels revealed additional protein bands which were not detected in the LC/MS analysis.

**TABLE 2:** Mass spectrometry analysis from Gel, results of purified Mip with coimmunoprecipitation

Protein	NCBI protein ID	Mass in kDa	Gene locus	FA 0%	FA 0,5%	Homology	Description
Mip	ABQ56418	24.849	Lpc_2500 975812-976513	+	+	FK506 Binding Proteins	Macrophage infectifity potentiator
SspB	ABQ54423	14.623	Lpc_0434 3231405-3231800	+	+	Stringent starvation proteins	Stringent starvation protein B ClpXP protease specifity enhancing factor
Lpc2061	ABQ55991	15.773	Lpc_2061 2911556-2911993	−	+	Homologs only in *Legionellaceae* family	Hypothetical protein Structural homology, AlphaFold 2.0 predicted model, to glycoside hydrolases according ProFunc database
FlaA	ABQ54733	47.858	Lpc_0756 1546346-1547773	−	+	Flagellin known as FliC in other organisms	Flagellin main flagellar monomer

A basic BLAST search using the NCBI database resulted with 67 homologous proteins to the hypothetical protein Lpc2061, 63 of which belonged to the *Legionellaceae* family. The other four hits were proposed to be hypothetical proteins from the Gammaproteobacteria Aquicella lusitana, Aquicella siphonis and Legionella gormanii. To gain further knowledge about the identified proteins and their putative functions, the ProFunc database was used. Lpc2061 showed homology to the glycoside hydrolase family. Taken together, the non-linking and cross-linking coimmunoprecipitation experiments with Mip revealed binding proteins of variable interaction strengths, which encompass diverse functional categories.

### Validation of Mip interaction partners and influence of FK506 on coimmunoprecipitation.

**FIG 2:**
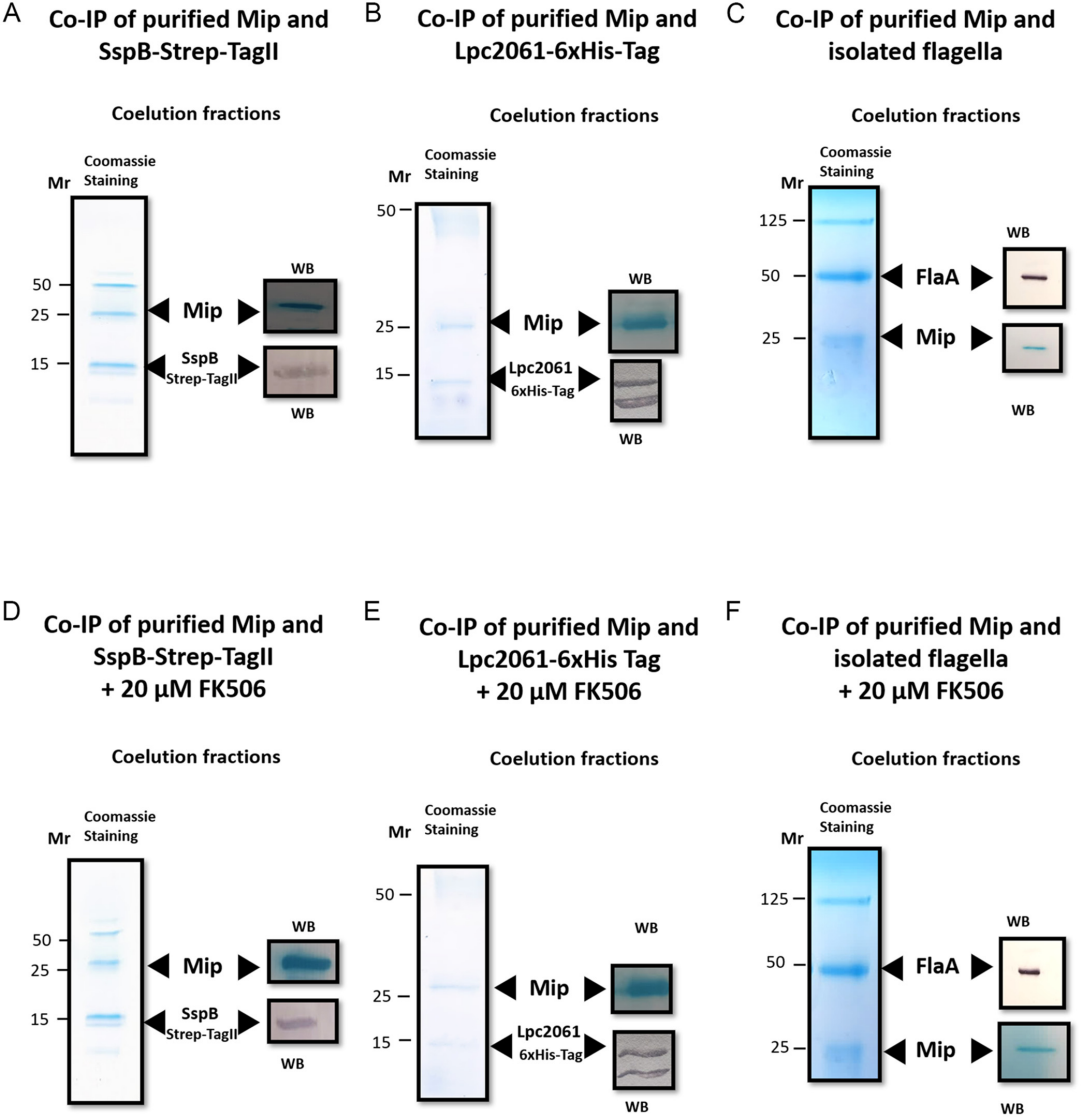
Validation of Mip interaction partners via the coimmunoprecipitation (CoIP) of SspB-Strep-TagII, Lpc2061-6xHis-Tag, and the purified native flagellin of L. pneumophila. Recombinant Mip was immobilized on magnetic DynaBeads coated with Protein G, 2D8, and 22/1 antibodies and was loaded with 1 mg of each purified interaction partner. All coeluates were resolved by SDS-PAGE and Coomassie-stained (left side of panels). The associations of the interaction partners were confirmed by Western-blotting (WB). (A) SspB-Strep-TagII was purified by a Strep-Tactin column. Western blots (WB) with anti-Strep and anti-Mip antibodies confirmed the interaction partners. (B) Lpc2061-6xHis-Tag was purified by a nickel column. Western blots (WB) with anti-6xHis and anti-Mip antibodies confirmed the interaction partners. (C) Coimmunoprecipitation of isolated FlaA and recombinantly produced Mip were confirmed by the anti-FlaA and anti-Mip antibodies. To analyze the influence of FK506 on the coimmunoprecipitation of Mip and the bacterial interaction partners, recombinant Mip was immobilized on magnetic DynaBeads coated with Protein G, 2D8, and 22/1 antibodies, incubated with 20 μM PPIase inhibitor FK506, and loaded with 1 mg of the purified interaction partners SspB-Strep-TagII, Lpc2061-6xHis-Tag, or native flagellin from L. pneumophila, respectively. (D) The coimmunoprecipitation of recombinantly produced Mip and SspB-Strep-TagII was not influenced by 20 μM FK506. Western blots (WB) with anti-Strep and anti-Mip antibodies confirmed the presence of the interaction partners. (E) Addition of 20 μM FK506 reduced the coimmunoprecipitation of Mip and Lpc2061-6xHis-Tag. Western blots (WB) with anti-6xHis and anti-Mip antibodies revealed a strong reduction of Lpc2061. (F) The coimmunoprecipitation of recombinantly produced Mip and isolated FlaA was not influenced by 20 μM FK506. Western blot (WB) with anti-FlaA and anti-Mip antibodies confirmed the presence of the interaction partners. For the uncropped Western blots, see Fig. S4.

To validate the identified interaction partners of Mip by a complementary approach, tagged variants of SspB and Lpc2061 were recombinantly produced in E. coli, whereas the native flagellin was purified from wild-type L. pneumophila (Table 1). Recombinant Mip was immobilized on magnetic beads coated with Protein G-antibodies as previously specified and then loaded with 1 mg of each purified interaction partner. The degree of association in terms of strength and stability displayed by each of the partners was relatively quantified with respect to band intensity in Coomassie-stained and Western blotted gels by using the ImageJ band intensity determination tools (Fig. 2A–F). When SspB was pulled down with immobilized Mip, a band at 15 kDa, along with Mip, appeared on the gel, which was further confirmed by Western blotting (Fig. 2A). The intensity of the SspB band on the PVDF membrane was the highest, with a relative value of 5028.832 intensity units (Table 3). Purified Lpc2061 coeluted with Mip resulted in a clear band at 15 kDa, which showed a positive signal for the 6×His-Tag in Western blotting (Fig. 2B). The band of Lpc2061 emitted a relative value of 4687.276 intensity units (Table 3). Besides Lpc2061, Mip was successfully copurified as a 25 kDa band, and this was detected in the Western blot. When isolated flagella were added to immobilized Mip, clearly visible bands were detected at about 50 kDa and 25 kDa which correspond to FlaA and Mip, respectively (Fig. 2C). The intensity of the band from the Western blotting was, at 4314.004 units, the lowest compared to the other partners (Table 3). Since the antibodies used for the coimmunoprecipitation showed no signal for the purified interaction partners, the coelution is regarded as specific to the interaction with Mip (Fig. S3 and S4). These data confirm the interactions of Mip with the respective proteins, with SspB being the strongest binder, followed by Lpc2061 and FlaA.

**TABLE 3:** Quantification of band intensities from the coelution fractions via ImageJ

Coeluted with Mip	Quantification of antibody signal from Western blot in relative units
SspB-Strep-TagII 15 kDa	5028.832
Lpc2061-6xHis-Tag 15 kDa	4687.276
FlaA 50 kDa	4314.004
SspB-Strep-TagII + 20μM FK506 15 kDa	5181.741
Lpc2061-6xHis-Tag + 20μM FK506 15 kDa	4523.134
FlaA + 20μM FK506 50 kDa	4378.189
Coeluted with Mip	Quantification band intensities in Coomassie stained gels in relative units
SspB-Strep-TagII 15 kDa	4297.674
Lpc2061-6xHis-Tag 15 kDa	1904.648
FlaA 50 kDa	5678.152
SspB-Strep-TagII + 20μM FK50615kDa	4235.988
Lpc2061-6xHis-Tag + 20μM FK506 15 kDa	794.87
FlaA + 20μM FK506 50 kDa	5743.463
Coeluted with Mip	Quantification band intensity at 15 kDa in Coomassie stained gels in relative units
Flagella + FK506 + Lpc2061	4603.669
Flagella + Lpc2061	7386.962

Since Mip is a FK506 binding protein (FKBP), we also assessed whether FK506 is able to prevent the binding of SspB, Lpc2061, and FlaA to recombinant Mip. Hence, we performed the aforementioned coimmunoprecipitation approach by adding 20 μM FK506. Since FK506 binds to the C-terminal binding pocket of Mip (24, 25), this approach also identifies whether or not this pocket is involved in the binding of the identified protein interaction partners. Each of the pulldown assays of the individual partners exhibited both proteins, Mip and the partner, in the eluates (Fig. 2D–F). However, a significant decrease of the hypothetical protein Lpc2061 was detected when the immunosuppressive drug was added (Table 3). These data suggest that the Mip-Lpc2061 interaction requires the C-terminal PPIase domain of Mip.

### The dimerization region and Y185 of Mip are required for the binding of Lpc2061 and FlaA.

**FIG 3:**
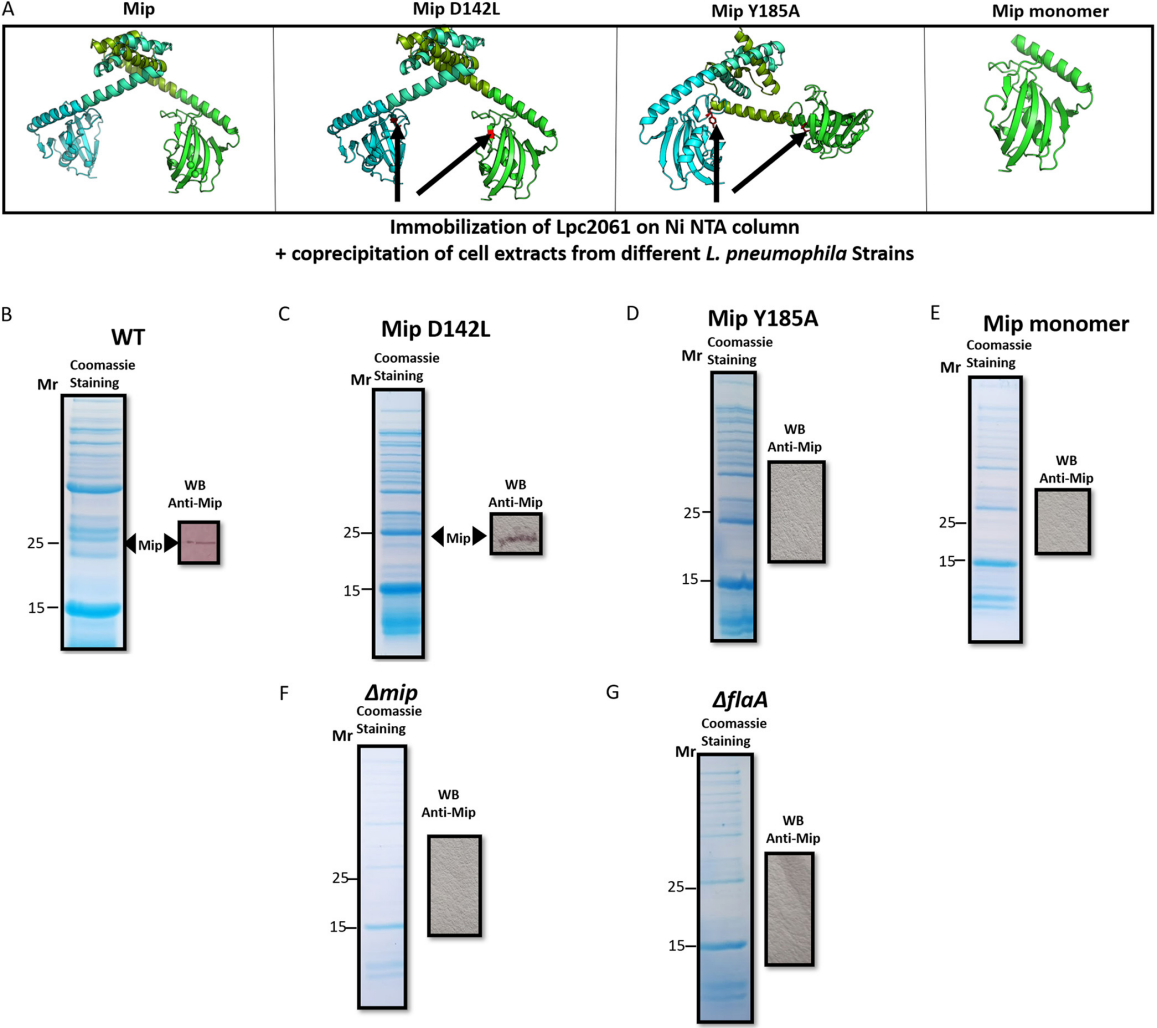
Coprecipitation of 6xHis-tagged Lpc2061 immobilized on nickel columns and Mip variants with single amino acid substitutions or an N-terminally truncated Mip monomer. (A) The respective structure images of L. pneumophila wild-type Mip with 100% PPIase activity, MipD142L with 6.2% PPIase activity, MipY185A with 2% PPIase activity, and monomeric Mip^(77-213)^ with 100% PPIase activity were generated using the PyMol tools. Site specific mutations are highlighted in red. Heterologously produced 6xHis-tagged Lpc2061 was incubated with whole-cell extracts from L. pneumophila strains expressing the respective Mip variants. Coeluates were analyzed by SDS-PAGE and Coomassie staining. Immunodetection on Western blots (WB) with anti-Mip antibodies were used to determine the binding capacities of the different Mip variants to Lpc2061. (B) Wild-type Mip and (C) Mip D142L were able to bind Lpc2061. (D) Mip Y185A and (E) monomeric Mip^(77-213)^ were unable to bind Lpc2061. (F) The whole-cell extract of L. pneumophila *Δmip* was used as a negative-control. (G) Mip was not detected after using whole-cell extracts from L. pneumophila *ΔflaA* for coprecipitation, suggesting that FlaA reinforces the Mip-Lpc2061 interaction. For the uncropped Western blots, see Fig. S5.

To further specify the interaction of Mip with the respective binding partners, we utilized L. pneumophila strains expressing Mip variants with single amino acid substitutions or an N-terminally truncated Mip^(77-213)^ monomer (Table 1). L. pneumophila JR32-2.2 (Mip^(Y185A)^ with 2% PPIase activity), JR32-2.3 (Mip^(D142L)^ with 6.2% PPIase activity), and JR32-2.4 (monomeric Mip^(77-213)^ with 100% PPIase activity) were used (16, 19). All of the versions of Mip are depicted in Fig. 3A. Heterologously-produced, His-tagged Lpc2061 was retained within a nickel-NTA affinity column, which also resulted in additional bands (Fig. S3A), as in the coimmunoprecipitation profile in the cross-linked samples (see Fig. 1B for comparison). Whole-cell extracts derived from the L. pneumophila strains expressing the respective Mip variants were incubated with immobilized recombinant Lpc2061. After stringent washing, the coeluates were analyzed by SDS-PAGE and immunodetection using anti-Mip antibodies to monitor the binding of the different Mip variants to Lpc2061 (Fig. 3B–F; Fig. S5A–D). While the Mip^(D142L)^ variant, copurified along with Lpc2061, Mip^(77-213)^ and Mip^(Y185A)^ were unable to bind Lpc2061. Moreover, additional proteins bound to Lpc2061 (besides Mip), and these varied depending upon the Mip variant used (Fig. 3 B–G; Fig. S3A). Wild-type Mip and Mip^(D142L)^ showed more intense bands compared to Mip^(Y185A)^, monomeric Mip^(77-213)^, and whole extracts of L. pneumophila Δ*mip*, indicating that the Mip-Lpc2061 interaction is responsible for the observed additional protein binding.

Because of the possible colocalization on the cell surfaces of Lpc2061 (predicted with SignalP) (29) and FlaA (30), we also examined a possible Mip:FlaA:Lpc2061 interaction. To assess this, first, whole-cell extracts isolated from the L. pneumophila *flaA*-negative mutant were added to likewise immobilized Lpc2061. In this scenario, Mip was neither detected on the SDS-PAGE nor in the Western blot (Fig. 3G; Fig. S5E). This suggested that FlaA reinforced the Mip-Lpc2061 molecular interaction. In a subsequent experiment, isolated flagella (1 mg/mL) from the L. pneumophila wild-type strain were added to immobilized Lpc2061 that was equally incubated with the L. pneumophila *flaA*-negative mutant whole-cell extract, as specified in the previous assay. Consistently, Mip-Lpc2061 coelution was fully restored by adding purified FlaA to the sample (Fig. S5F and G). Due to the observed effect with the L. pneumophila *flaA*-negative mutant, another coimmunoprecipitation was performed by adding both isolated flagella and Lpc2061 protein to immobilized recombinant Mip protein. Mip was coeluted with both of the proteins, and the presence of flagella resulted in an enhanced detection signal of Lpc2061 (Table 3; Fig. S5H–I). The immobilization of SspB resulted in the binding of all tested Mip variants. The SspB-Mip binding was not dependent on FlaA (Fig. S5J). In conclusion, the dimerization region of Mip and amino acid Y185 appear to be crucial for the interaction between Lpc2061 and Mip, which is strengthened by FlaA, while the binding of SspB to Mip occurred, irrespective of the tested variant, and was not affected by the presence of FlaA.

### Modeling of interaction partners and global docking with Mip suggests nonoverlapping binding interfaces.

**FIG 4:**
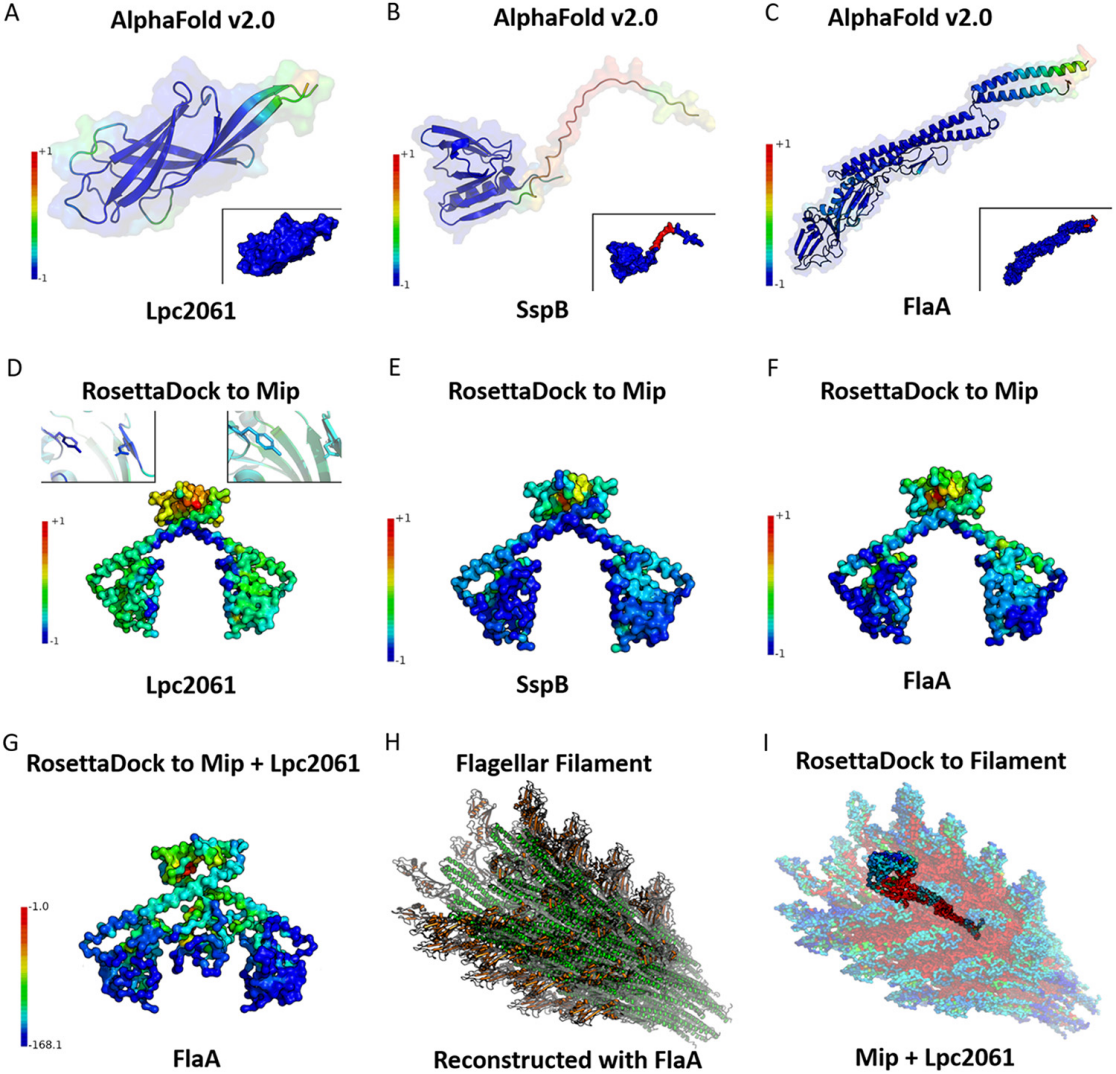
Modeling of the interaction partners of Mip with AlphaFold v2.0 and of the global docking of each structure to Mip using RosettaDock. The prediction scores were normalized to a range from −1 to +1 (best to worst). The disorder classifications on the protein surface plot are color coded (blue for a strong binding possibility and red for a weak binding possibility). Accordingly, the structure predictions for (A) Lpc2061, (B) SspB, and (C) FlaA are illustrated with color coded modelling scores. The insets show disordered regions, which are classified by pLDDT <50. (D) The global docking of Lpc2061, (E) SspB, and (F) FlaA to Mip identifies nonoverlapping binding interfaces. For visualization, the surface of Mip was colored per residue by the interface score of the best poses in which the docking partner has contact. The docking scores were normalized by minimum and maximum values of the respective ensemble in the interval from the best interface score (−1) to the worst interface score (+1). (G) The docking site of FlaA on a refined version of the best Mip-Lpc2061 pose. The normalized interface score is color coded on Mip-Lpc2061. To better visualize the difference an exponential scaling law $-e^{-0.1\cdot I_{\mathrm{sc}}}$ was applied to the interface score. (H) Flagellar filament reconstructed from the FlaA structure prediction and the Cryo-EM structure of P. aeruginosa flagellar filaments (PDB ID: 5WK6). L. pneumophila specific FlaA regions unresolved in the Cryo-EM structure are highlighted in orange. (I) Global docking of the modeled flagellar filament surface, Mip, and Lpc2061.

As no experimentally determined complete structures exist for the identified interaction partners, we predicted these *in silico* using AlphaFold v2.0 (Fig. 4). The predicted structure of Lpc2061 consists of β-sheets connected by loop regions, with a good score, overall (normalized score around 0), except for some surface loop regions (amino acids 16 to 21 and 49 to 54) as well as the N and C termini (Fig. 4A). The inset of Fig. 4A shows that there is likely no disorder in this structure. The SspB model, according to AlphaFold v2.0, shows a structure that is similar to the resolved parts of the E. coli SspB 1TWB (31) (Fig. 4B). The tail-like structure of L. pneumophila SspB harbors a region that is classified as disordered (inset of Fig. 4B). FlaA is predicted to be a long protein which can be separated into a complex region that contains α-helices as well as β-sheets and loops as well as a long domain that primarily contains α-helices (Fig. 4C). The only regions classified as possibly disordered are small regions at the N and C termini (inset of Fig. 4C).

**FIG 5:**
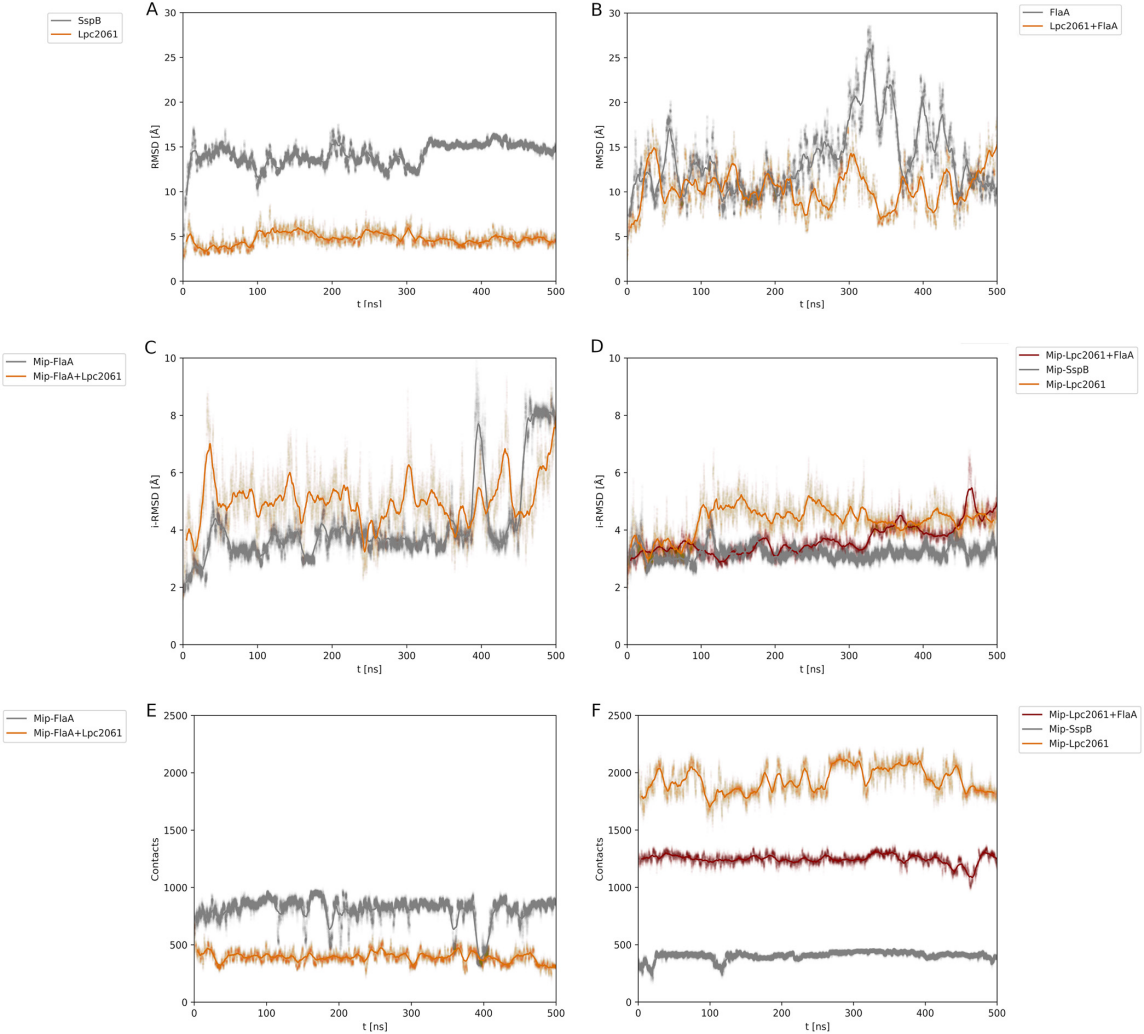
MD simulations of the best global poses of SspB, Lpc2061, and FlaA with Mip. The RMSDs were plotted over a simulation time of 500 ns and (A) revealed a stable interaction for SspB and Lpc2061. (B) Simulations for FlaA and Lpc2061 with FlaA, respectively, revealed strong deviations. However, the addition of Lpc2061 reduced the fluctuations. (C) The iRMSD of the Mip-FlaA interface score exhibited higher peaks, and these were not reduced by the addition of Lpc2061. (D) The stability of Mip-Lpc2061 was increased by the addition of FlaA and remained on a similar level to that of the Mip-SspB interface. (E) The number of contacts, defined as a 10 Å vicinity of atoms within the protein-protein interface, between Mip and FlaA over the duration of the simulation decreased when Lpc2061 was added. (F) Mip-SspB showed the fewest contacts, followed by Mip-Lpc2061 plus FlaA and Mip-Lpc2061.

To develop a hypothetical model for the three interaction partners, we ran global docking simulations with Mip (Fig. 4D and E). Unanimously, the worst overall docking scores are located in the upper part of the Mip dimerization region. For Lpc2061, the best scoring regions are inward of the C-terminal PPIase domain and at the basis of the α-helix above the dimerization region (Fig. 4D). Furthermore, there was a visible difference in the interface scores of the residues Asp142 and Tyr185 in each PPIase domain. The normalized interface on the left PPIase domain was −0.79 for Asp142 and −0.98 for Tyr185, whereas it was −0.4 for Asp142 and −0.59 for Tyr185 on the right PPIase domain. For SspB, there seems to be no clear optimal area standing out from the ensemble. Mostly, both PPIase domains of Mip showed a low interface score, which suggests that this protein has no specific binding site for SspB (Fig. 5E). For FlaA (Fig. 5F), the region containing the lowest interface score was located more on the outward facing part of the PPIase domain (residues 96 to 106). Residues 107 to 212 of Mip, on the other hand, are closer to the long α-helices (residues 55 to 96), which connect the PPIase regions with the dimerization interface of the homodimer. Since the putatively best binding region for FlaA is at a different nonoverlapping location, compared to the low interface score region identified for Lpc2061, both score distributions seem to be complementary. This is in accordance with the observation that FlaA reinforces the Mip-Lpc2061 interaction. This tripartite interaction was modeled by docking FlaA to the refined best pose of the Mip-Lpc2061 interaction. As illustrated by the color code of the normalized interfaces after applying the exponential scaling law*-e*^−0.1×^*^I_SC^* (32, 33) to better visualize the differences (Fig. 4G), the tripartite ensemble shows similar regions of the most negative scores compared to the respective single interactions. The complete histograms of the docking interface scores of Lpc2061, SspB, and FlaA, as well as those of the whole ensemble (Mip with Lpc2061 and FlaA) further illustrates this view (Fig. S6).

For a further interpretation of our data set, we also modeled the flagellar filament by fitting multiple copies of the predicted L. pneumophila FlaA structure into the near-atomic resolution cryo-EM structure (PDB ID: 5WK6) of Pseudomonas aeruginosa PAO1 (34). The reconstructed filament structure is illustrated in Fig. 4H, in which the L. pneumophila specific regions of FlaA are highlighted. Via the global docking of the modeled filament surface, Mip and Lpc2061, we generated 8,000 poses (Fig. 4I). The best pose of FlaA, Mip, and Lpc2061 in this filament is consistent with the global docking of the tripartite interactions, independent of the filament context. Moreover, the L. pneumophila specific regions in the filament revealed the best binding interfaces (lowest scores) for binding Mip and Lpc2061.

### Molecular dynamic simulations predict increased stability for the tripartite interaction of Lpc2061, Mip, and FlaA.

Based on our biochemical results, we further hypothesized that the tripartite interaction of Lpc2061, Mip, and FlaA entails increased stability. Molecular dynamics (MD) simulations starting from the refined docking of the best global poses with Mip are illustrated in Fig. 5. For the entire structures of SspB, Lpc2061, FlaA, and Lpc2061 with FlaA, respectively, the overall RMSD were plotted over a simulation time of 500 ns (Fig. 5A and B). The interface RMSD (i-RMSD) and contacts for the interface atoms of the respective Mip-interaction partners were similarly plotted over the simulation time (Fig. 5C–F). The RMSDs of the overall simulations were used to evaluate the stability of the observed dynamics. Simulations for Mip interacting with SspB or Lpc2061 revealed a relatively stable interaction, since a horizontal line with relatively little perturbations represents a largely constant value (Fig. 5A). The simulations of FlaA or Lpc2061 with FlaA exhibited far more deviations (Fig. 5B). FlaA alone was unstable between 300 ns and 450 ns, and the addition of Lpc2061 to FlaA showed increased stability after 200 ns, although fluctuations still occurred. A similar trend was observed with i-RMSD, with which the Mip-FlaA interface score exhibited more and higher peaks compared to the Mip-FlaA interface score when Lpc2061 was added (Fig. 5C). The i-RMSD of the Mip-SspB interface revealed a higher stability compared to the Mip-Lpc2061 interface. However, the stability of the Mip-Lpc2061 was increased by the addition of FlaA and was then on a similar level as the Mip-SspB interface (Fig. 5D). Interestingly, the absolute number of contacts between Mip and FlaA over simulation time decreased when Lpc2061 was added (Fig. 5E). Mip-SspB showed the fewest contacts, followed by Mip-Lpc2061 plus FlaA and Mip-Lpc2061 (Fig. 5F). Nevertheless, the tripartite interaction appeared more stable, as large peaks were absent. All four simulations are illustrated in Supplemental Movies M1–M4. The minimum RMSD between each pose from the flagellar filament and the MD trajectory for Mip-Lpc2061 plus FlaA scattered around the interface score are illustrated in Fig. S7. The interaction of Lpc2061, however, seems to be stable, judging from both the overall as well as the interface RMSD values. This is also visible during the 500 ns trajectory, as visualized in Supplemental Movie M1. Moreover, there is almost no deviation from the positioning of Lpc2061 between the two PPIase domains. The best pose was identical with the lowest RMSD in the trajectory. This is also illustrated in Supplemental Movies M5–M8, in which the MD trajectory is superimposed on the best poses of FlaA.

### Mip promotes flagellation and the yield of L. pneumophila FlaA.

As published previously, the formation of intact flagella correlates with the flagellin expression of L. pneumophila (35). To analyze whether Mip impacts bacterial motility, we first inspected L. pneumophila cells expressing wild-type Mip and Mip variants with single amino acid substitutions and the N-terminally truncated Mip monomer via phase-contrast microscopy. The L. pneumophila Δ*flaA* mutant, which is unable to produce FlaA or flagella, was used as a negative-control. This approach revealed that the motility of the L. pneumophila Δ*mip* mutant and cells expressing the monomeric Mip^(77-213)^, or Mip^(Y185A)^ was impaired (Movie movS9–movS13). The strain expressing Mip^(D142L)^ with 6.2% PPIase activity showed a slightly reduced motility compared to the L. pneumophila wild-type strain (Movie movS14).

**FIG 6:**
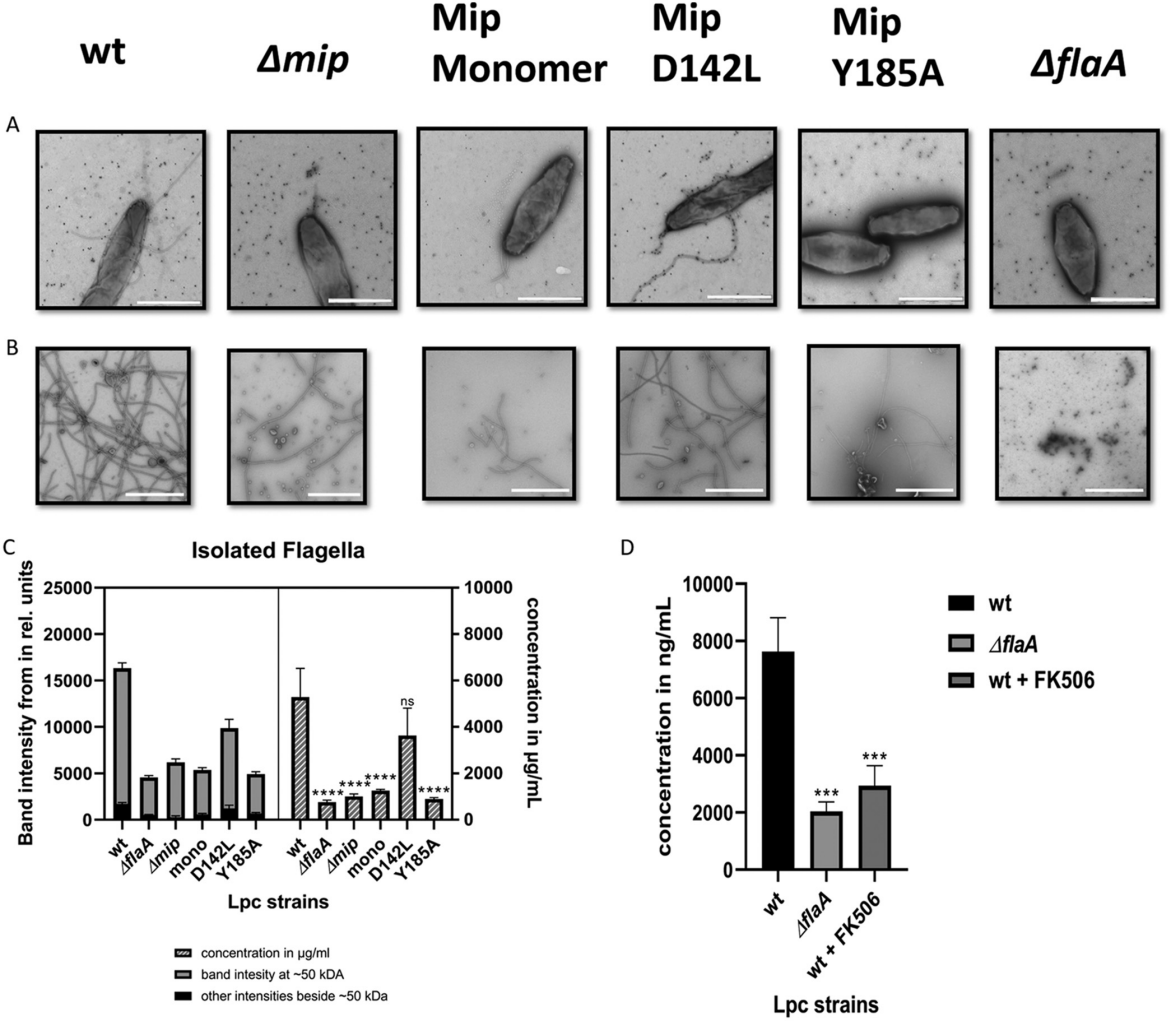
Impact of Mip on the bacterial flagellation and the quantification of FlaA. (A) TEM of isolated flagella from equal amounts of biomass of the L. pneumophila wild-type, the *Δmip* mutant, and strains expressing the N-terminally truncated Mip monomer and Mip variants with single amino acid substitutions (Mip D142L, Mip Y185A). The L. pneumophila Δ*flaA* mutant, which is unable to produce FlaA or flagella, was used as a negative control. Size: bar = 1 μm. (B) TEM of flagella isolated from 10^14^ bacteria of the respective L. pneumophila wild-type strain and mutants. (C) Quantifications of FlaA from Coomassie stained SDS gels (band ~50 kDa) were performed by using the ImageJ band intensity determination tools and Roti Nanoquant at 590 nm/450 nm. The identity of FlaA was confirmed by Western blotting (Fig. S8). L. pneumophila *ΔflaA* was used as a negative-control and was taken as the background for the quantification of FlaA. (D) Comparison of flagellar preparations from 10^11^ bacterial cells of the L. pneumophila wild-type, the *ΔflaA* mutant, and the wild-type treated with 20 μM FK506. Three replicates were compared via Dunnett’s multiple-comparison test. **, *P* < 0.01; ***, *P* < 0.001; ****, *P* < 0.0001; ns, not significant.

Since the monopolar flagella of L. pneumophila cells easily break under shear forces, which causes difficulties in quantifying them via TEM, we isolated flagella from equal amounts of biomass of the L. pneumophila wild-type strain and mutants (Fig. 6A & B). The TEM revealed intact flagella in preparations from the L. pneumophila wild-type strain and, to a much lesser extent, in the isogenic Δ*mip* mutant. Preparations from the Δ*flaA* mutant were completely devoid of flagellar structures. Preparations from L. pneumophila mutants expressing Mip-variants with reduced PPIase-activity or the N-terminally truncated Mip monomer also exhibited fewer flagellar structures, compared to the wild-type strain. Subsequent quantification of the 50 kDa FlaA protein by SDS-PAGE, Coomassie-staining, and ImageJ confirmed this observation (Fig. 6C). Again the L. pneumophila Δ*flaA* mutant was used as a negative-control, and the faint band intensity of 4031.3 units that resulted from the preparation of this mutant was taken as the background. The band intensities of the Coomassie-stained SDS gels revealed that the signal of FlaA extracted from the L. pneumophila wild-type strain was 10,589 units (Fig. 6C and Fig. S8). Strikingly, the flagellar preparations from the *mip*-negative mutant resulted in a band intensity of 1,889 units, a 5.6-fold decrease compared to that of the wild-type strain. The site-specific mutant expressing the Mip^(D142L)^-variant with 6.2% PPIase activity exhibited a significantly higher band intensity in Coomassie-stained SDS gels and a higher FlaA concentration (μg/mL) compared to the strains expressing Mip^(Y185A)^ (with 2% PPIase activity) or the monomeric Mip^(77-213)^ (with 100% PPIase activity) (Fig. 6C; Table S1 and S2). Similarly, treating L. pneumophila with 20 μM FK506 resulted in a lower FlaA yield (Fig. 6D; Table S3 and S4).

## DISCUSSION

PPIases of pathogens are involved in a broad spectrum of phenotypes, including virulence, metabolism, and multiple stress responses (36–42). Likewise, the PPIase Mip of L. pneumophila contributes to infection, collagen binding, phospholipase C-like activity, transmigration across tissue barriers, nematode colonization, surface translocation, and growth at suboptimal temperature (28, 43–45). Mip targeting antimicrobial inhibitors, such as cycloheximide, pipecolic acid, and FK506 derivatives further corroborated the observation of the moonlighting activities of Mip in the fundamental processes of infection (39, 46–50). Nevertheless, the natural substrates and specific molecular activities of Mip remain largely elusive.

Here, we present for the first time the identification of bacterial interaction partners of L. pneumophila Mip. Immunoprecipitation revealed SspB (LPC_0434) as an *in vivo* interaction partner of Mip. Cross-linking with FA additionally identified the hypothetical protein Lpc2061 and the main flagellar monomer FlaA (LPC_0756) as interaction partners. The SspB homologues (e.g., those in E. coli [52% identity] and *Pseudoalteromonas* spp. [62% identity]) are well-known dimeric adaptor proteins which increase the rate at which ssrA-tagged substrates are degraded by tethering them to the ClpXP protease (51, 52). Interestingly, the SspB of Salmonella enterica serovar Typhimurium, which, like L. pneumophila, has no secretion signal, has been found in bacterial culture supernatants and is inserted into host cell plasma membranes shortly after bacterial infection (53). The ClpXP protease of this pathogen negatively regulates flagellar synthesis. The respective ClpXP deletion mutants exhibit overproduction of the flagellar protein, a 4-fold increase in the rate of transcription of *fliC*, and a hyperflagellated phenotype (54). Whether Mip intercepts SspB and by this means downregulates the ClpXP-dependent repression of the flagellar regulon in L. pneumophila has yet to be shown. The hypothetical protein Lpc2061 shows structural homology to glycoside hydrolases; however, its actual function remains unclear. L. pneumophila FlaA is the major component of the surface exposed flagellar filament. Like SspB, FlaA is expressed during starvation periods in the postexponential phase of L. pneumophila (30).

The interactions with Mip were validated via a complementary approach using the tagged variants of SspB and Lpc2061 as well as purified native L. pneumophila FlaA. Moreover, the respective binding strengths, with SspB as the strongest, followed by Lpc2061 and FlaA, were semiquantified by determining the band intensities of Western blots. Since the PPIase activity of Mip is inhibited by FK506 (23), and since this macrolide decreased the coimmunoprecipitation of the hypothetical protein Lpc2061 and the binding between Mip and SspB was not affected by point mutations which reduce the PPIase activity, we conclude that the C-terminal PPIase domain of Mip is involved in the binding of Lpc2061. As several of the virulence and fitness functions of Mip are not related to enzymatic catalysis, but rather to moonlighting activities in the host (28, 47, 48), it is unsurprising that not all of the interactions were negatively influenced by FK506. Further characterization of the interactions by L. pneumophila strains expressing Mip variants with single amino acid substitutions or N-terminally truncated monomers revealed that the dimerization region and the amino acid Y185 of Mip are essential for the binding of Lpc2061, whereas these regions are not required for SspB binding. Previous studies already revealed that, mediated by a hinge in the long α-helix, both of the PPIase domains of dimerized Mip are subject to large fluctuating movements, allowing for the flexible cooperative binding of potential target structures (25). To what extent this applies to Lpc2061 remains to be clarified.

Interestingly, FlaA reinforced the Mip-Lpc2061 interaction, as adding isolated FlaA restored the coelution of Mip-Lpc2061 from a L. pneumophila *flaA*-negative mutant. This effect of FlaA was not observed for the Mip-SspB interaction. These experimental findings are in good agreement with computational modeling of the interaction partners and the global docking with Mip, since the putatively best binding region of FlaA shows no overlap with the binding interface of Lpc2061. Although the combination of 3D structure prediction and docking has a limited accuracy, MD simulations suggest an increased stability for the tripartite interaction of the Lpc2061-Mip-FlaA ensemble. Except for SspB, which could be coimmunoprecipitated without FA cross-linking and revealed the highest binding strength, a far-reaching consistency of our biochemical data and computational predictions regarding the stability of the interaction partners was observed. According to the best poses (docking), our computational data (MD) suggest that the interactions Mip-Lpc2061, Mip-SspB, Mip-FlaA, and Mip-Lpc2061-FlaA are stable. For the Mip-Lpc2061 interface in the tripartite complex, we see a lower i-RMSD. Also, the fluctuations in the number of contacts are less in the tripartite complex compared to the Mip-Lpc2061 interaction. This indicates that the Mip-Lpc2061 interface in the tripartite complex is more stable than the Mip-Lpc2061 binary interaction. Since SspB showed no outstanding specific binding area in the global docking with Mip (which is consistent with our biochemical data) and molecular dynamics simulations using the best pose with Mip, the discrepancy regarding the best binder can be explained. Overall, the results from biochemical assays, modeling, docking, and dynamic simulations are consistent, help to portray the putative binding interfaces, and suggest an increased stability for the tripartite interaction of the ensemble of Mip, Lpc2061, and FlaA.

To better understand the functional implications of the observed protein interactions and the hypotheses deduced from the computational biology, we microscopically analyzed flagellar preparations of the L. pneumophila wild-type strain, the isogenic Δ*mip* mutant, and the L. pneumophila strains producing Mip variants with single amino acid substitutions or N-terminally truncated monomers of Mip. Moreover, we quantified FlaA preparations of these strains. We clearly demonstrated that wild-type Mip promotes the flagellation of L. pneumophila and the yield of FlaA. Interestingly, all of the L. pneumophila strains that were expressing Mip variants were significantly less flagellated compared to the wild-type strain. In accordance with the biochemical results showing that FlaA and Lpc2061 mutually reinforce their binding to Mip, which was also suggested by the MD simulations, we observed that the binding regions of Mip in the Mip-Lpc2061 interaction positively influences flagellation. The L. pneumophila mutants expressing the Mip^(Y185A)^ or the monomeric Mip^(77-213)^ variant, which bind less Lpc2061, were less flagellated and yielded less FlaA. Also, FK506 treatment resulted in a lower FlaA yield. Thus, we propose a model in which Mip in concert with Lpc2061 is important for L. pneumophila flagellation.

In L. pneumophila, the expression of a single monopolar flagellum is life cycle-dependent and correlates with the virulent postexponential growth phase. The pathogen is not flagellated during intracellular replication but becomes so during the transmissive phase, when it lyses its spent host and searches for a new replication niche (55). Then, like Mip, the major subunit FlaA positively affects the early phase of infection of eukaryotic host cells (35), as the non-flagellated mutants of L. pneumophila are less infective for amoebas and macrophages (35, 56). Moreover, FlaA mediates the toxicity to host cells (30, 57). We hypothesize that certain effects of Mip or FlaA on L. pneumophila pathogenicity are mediated or regulated by their interaction. It is even imaginable that Mip assists in the flagellar assembly or regulation, as is suggested by our flagellation assays. Interestingly, monomeric FlaA induces innate the immunity of alveolar macrophages and lung epithelial cells (30). The globular domain D_1_ and the D_0_ polymerization domain of FlaA are recognized by TLR5, which plays an important role during bacterial clearance (58, 59). Accordingly, a tight control of flagellation and an extracellular release of FlaA appear to be critical for the environmental fitness and pathogenicity of L. pneumophila.

To the best of our knowledge, this is the first report in which a bacterial PPIase was demonstrated to influence flagellation. Our results revealed Mip as a binding partner of FlaA and as an amplifier of L. pneumophila flagellation. Our results point toward a positive modulation of this phenotype by the Mip interaction partner Lpc2061, a protein of yet unknown function. Moreover, starvation signals are also known to play a role in the flagellar regulon via ppGpp (30). Whether or not (and if yes, in which way) the Mip binding partner SspB is also involved in this interaction remains elusive and should be addressed in further studies.

## MATERIALS AND METHODS

### Bacterial strains and culture.

L. pneumophila strains were grown either on Buffered Charcoal Yeast Extract (BCYE) agar (10 g/L yeast extract and 10 g/L ACES (N-(2-Acetamido)-2-aminoethanesulfonic acid) buffer [pH 6.9] supplemented with 0.4 g/L l-cysteine, 0.25 g/L iron (III) nitrate, and 15 g/L agar) or in Buffered Yeast Extract (YEB) medium (10 g/L yeast extract, 10 g/L ACES buffer [pH 6.9] supplemented with 0.4 g/L l-cysteine and 0.25 g/L iron (III) pyrophosphate). Luria-Bertani (LB) medium (5 g/L yeast extract, 10 g/L peptone, 5 g/L NaCl) was used for culturing the E. coli strains. When required, antibiotics or FK506 were added to the following final concentrations: 20 μg/mL kanamycin, 12.5 μg/mL chloramphenicol, 50 μg/mL streptomycin, and 20 μM FK506 for L. pneumophila and 100 μg/mL ampicillin for E. coli. The bacterial strains and primers employed throughout this work are listed in Table 1.

### *In vivo* cross-linking and cell fractionation of L. pneumophila.

L. pneumophila was grown in 1 L YEB to an OD_600_ (optical density) of 1.8. Different concentrations of formaldehyde (vol/vol) were added to the culture medium: 0%, 0.25%, 0.5%, 0.75%, and 1%. formaldehyde (FA) was allowed to penetrate bacterial membranes and elicit cross-linking reactions for 30 min at 37°C with shaking at 200 rpm. The reactions were quenched with 130 mM glycine for 5 min at 200 rpm. After this, the cells were harvested by centrifugation at 4,000 × *g* for 20 min at 4°C. From here on, the protocol was carried out at 4°C. The pellet was resuspended in suspension/lysis buffer (100 mM Tris-HCl [pH 7.5], 150 mM NaCl, 1 mM EDTA). A tablet of protease cocktail inhibitor (Roche) was added per 10 mL volume. Bacteria were subsequently homogenized with FastPrep (MP Biomedicals FastPrep-24) (two times, 6 m/s for 30 s). Centrifugation at 4,000 × *g* for 20 min followed in order to eliminate cell debris. The supernatants were ultracentrifuged for 1 h at 100,000 × *g*. Subsequently, the insoluble fraction was dissolved in PBS-buffer supplemented with 1.8% (vol/vol) Triton X-100 overnight at 4°C under continuous rotation. Thereafter, the solubilized mix was centrifuged at 16,000 × *g* for 20 min, and soluble fractions were harvested. The isolated protein fractions were immunoprecipitated (see “Immunoprecipitation of Mip and analysis of binding domains”), separated by SDS gel electrophoresis, and stained and/or transferred for Western blotting (60).

### Recombinant protein production.

The genes of the putative interaction partners *lpc2061* and *lpc0434* (SspB) were amplified from the L. pneumophila genome using the primers specified in Table 1. All primers contained restriction sites as overhangs. *lpc2061* and *lpc0434* were treated with corresponding restriction enzymes and ligated into the equally treated expression vectors pET-22b(+) (Novagen 69744-3) or pET-52b(+) (Novagen 71554), respectively. The resulting constructs were named p2061-His and pSspB-Strep. Plasmids were introduced into chemically competent E. coli BL21 cells (61). This led to the construction of the recombinant strains BL21-pet22b-*lpc2061* and BL-21-pet52b-*sspB*. The most suitable conditions for Lpc2061-6×His-Tag production turned out to be 1 μM IPTG induction and incubation at 17°C overnight. Recombinant SspB-Strep-TagII production was successful upon induction with 1 μM IPTG and incubation at 37°C for 6 h. The bacteria were harvested and washed twice with PBS (4,000 × *g* for 20 min at 4°C). Thereafter, the pellets were resuspended in 20 mL PBS supplemented with 1 tablet of protease inhibitor cocktail (Roche), wherein 10 mM imidazole was added to the buffer for Lpc2061. Then, the mixture was processed with a FrenchPress (ThermoFisher FA-032) and centrifuged again at 4,000 × *g* for 20 min at 4°C. The supernatants were filtered through a 0.45 μm filter, and the resulting extracts were applied to a nickel-NTA column (Macherey-Nagel number 745415.5) or a StrepTactin column (Iba Life Sciences number 2-5998-000) for Lpc2061 and SspB, respectively. Purification was performed as specified by the manufacturers. Flowthrough, wash, and elution fractions were collected for further analyses via SDS gel electrophoresis and Western blotting.

### Native isolation of flagella.

L. pneumophila strains were grown in YEB medium overnight, and 200 μL of cultures were plated on BCYE agar. The BCYE plates were incubated at 30°C for 5 days, and 10^11^ (for the inhibitor experiments) and 10^14^ bacteria (for the Mip variant experiments) were resuspended in 20 mL PBS. The suspensions were then blended with a homogenizer (Polytron PT2500 E) for 3 min. Centrifugation was subsequently performed at 16,000 × *g* for 15 min at 4°C. The supernatants were withdrawn and centrifuged again at 40,000 × *g* for 3 h at 4°C. The resulting pellets were pulled out and resuspended in a 5 mL final volume of phosphate buffer, and isolates were diluted to 2 mg/mL total protein content (62). The isolation of flagella from equal amounts of bacteria (10^11^ or 10^14^ bacteria) were performed in duplicate and repeated 3 times. The concentrations were measured with a Roti Nanoquant (Carl Roth GmbH + Co., KG number K880) and determined according to OD at 590/450 nm. The purities of the flagella preparations were confirmed spectrophotometrically using a Nanodrop Implen NanoPhotometer N50 at 260/280 nm.

### Immunoprecipitation of Mip and analysis of binding domains.

To immunoprecipitate Mip, DynaBeads (Thermo, number 10003D) were coupled with 22/1 and 2D8 monoclonal mouse antibodies (63). L. pneumophila were grown in liquid culture and homogenized using FastPrep. When the solubilization of the membrane fractions was required, 1.8% (vol/vol) of Triton X-100 was added as described in “*In vivo* cross-linking and cell fractionation of L. pneumophila*”* (60). The resulting solubilized membranes were incubated with DynaBeads for the immobilization and purification of Mip from membrane fractions (28 kDa) or the respective variants. When required, 1 mg of recombinant or native proteins (see “Recombinant protein production” and “Native isolation of flagella”) were added to Mip immobilized on DynaBeads and were harvested as described above. Alternatively, recombinantly produced Mip (25 kDa) was used for coprecipitation procedures (15). The influence of FK506 (Sigma-Aldrich F4679) on interactions between Mip and the protein partners was analyzed by adding 20 μM PPIase inhibitor to DynaBeads-bound recombinant Mip (15). All of the coeluates, including the flowthrough and wash fractions (purity control) were resolved on a 15% acrylamide SDS-PAGE. The gels were Coomassie-stained (100 g/L (NH_4_)_2_SO_4_, 100 mL/L H_3_PO_4_, 20% [vol/vol] methanol) overnight, as described previously (64). Silver staining of the gels was also performed as described previously (65). Mip was detected employing rabbit anti-Mip polyclonal antibodies as specified elsewhere (66). In brief, proteins were transferred onto a PVDF-membrane via semidry blotting (5 min, 1.3 mA, and 25 V). The membranes were blocked with TBST (0.2% [vol/vol] Tween 20) containing 3% (wt/vol) low fat milk powder. Subsequently, TBST with 1% (wt/vol) milk powder, including the primary antibodies (1:10,000) for Mip (66) and FlaA detection (67) and the anti-Strep (Iba Lifescience Strep-Tactin AP conjugate 2–1503-001) and anti-His (Invitrogen 6×-His Tag Monoclonal Antibody) antibodies for the detection of SspB and Lpc2061, respectively, were used. Secondary mouse or rabbit antibodies were added at 1:10,000 dilution in 1% (v/w) TBST milk powder. Chromogenic reactions by which to visualize the respective proteins were carried out by adding 0.66% (vol/vol) Nitro Blue Tetrazolium (NBT) and 0.33% (vol/vol) 5-bromo-4-chloro-3-inodyl phosphate (BCIP). The quantification of proteins was performed using the ImageJ band intensity determination tools. The quantification reflects the relative amounts as a ratio of each protein band, relative to the lane’s loading control. For the LC-MS/MS analysis, the samples were migrated 10 cm into a SDS-PAGE and stained with Coomassie. The bulky band was excised with a scalpel and shipped to Proteome Factory AG for further analyses using a Thermo Scientific LTQ Orbitrap XL hybrid FTMS device in a medium gradient (see https://www.proteome-factory.com/ for further details).

### Motility and transmission electron microscopy of isolated flagella.

The motility of L. pneumophila strains and mutants was scored qualitatively by examining wet mounts of L. pneumophila broth cultures via phase-contrast microscopy at a magnification of 320×. A culture (stationary-phase) was defined as motile if at least half of the bacteria in a field of at least 100 cells were judged to exhibit rapid, directed movement. Movies of motile and nonmotile bacteria were recorded at a magnification of 320× using an Oppo-Find-X5-Pro Android camera. Flagella samples were prepared as described in “Native isolation of flagella,” and 200 μL aliquots were used for negative staining. A thin carbon film was floated on sample droplets (approximately 30 to 40 μL) to allow for the adhesion of flagella. After 1 min, a 300 mesh copper grid was placed on the film to take it off. The filmed grid was washed twice with distilled water and incubated for 1 min on a droplet of 4% (wt/vol) uranylacetate, and this was followed by the removal of excessive liquids and drying. Electron microscopy was performed with a Libra 120 Plus (Zeiss, Germany) at an acceleration voltage of 120 kV and with calibrated magnifications using the WinTEM/ITEM software package.

### Modeling of proteins with AlphaFold v2.0.

To predict the structures of the potential Mip-interaction partners, Lpc2061, SspB, and FlaA, the standalone version of AlphaFold v2.0 (https://alphafold.ebi.ac.uk/) in monomer mode was used (68). The visualization of quality and the final ranking of the model were achieved via the local difference test (pLDDT)-scoring, which is based on the Cα positions (68). Since a pLDDT score of <50 is associated with disordered regions (68), this classifier was evaluated for the final predictions. For visualization, the prediction score was normalized on a range from −1 to +1 (best to worst). The results of the disorder classification were then visualized on the protein surface plot in the according insets with blue for no disorder and red for disorder. As inputs for the modeling, the sequences ABQ55991, ABQ54733, and ABQ54423 from the NCBI Database were used for Lpc2061, FlaA, and SspB, respectively. As the interactions occur in membrane fractions, we scanned the sequences for cleavage sites using SignalP 6.0 (29). For Lpc2061, we identified a cleavage site at residue 24. Accordingly, the sequence starting from residue 25 of ABQ55991 was used for the modeling. Since no cleavage sites were found in FlaA or in SspB, the entire sequences were used as inputs. Protein visualizations were created using PyMol (32).

### Molecular docking.

To investigate the interactions of Lpc2061, SspB, and FlaA with Mip *in silico*, the global docking protocol from the Rosetta framework was used to generate an ensemble of 100k poses, each (33). This method fixes one protein and tries to randomly place the other mobile molecule on its surface so that the energy function is minimized. For visualization, the surface of Mip was colored per residue by the interface score of the best pose in which the docking partner had a contact with Mip. The docking scores were again normalized for visualization by the minimum and maximum values of the respective ensemble in the interval from best interface score (−1) to worst interface score (+1). The best interface score per residue over all of the poses was used for the surface coloring, which indicates good or bad contact sites with the interaction partners. The protein visualizations were created with PyMol (32).

### Molecular dynamics simulations.

Molecular dynamics (MD) simulations of the refined best pose from the global docking ensemble were performed in GROMACS (69) by using the CHARMM36 force field (70). After placing the molecule inside a dodecahedron with periodic boundary conditions, ions were added to neutralize the overall charge. After equilibration for 100 ps in the NVT [(number of particles) × (volume) × (temperature)] and then the NPT [(number of particles) × (pressure) × (temperature)] ensembles, the production run was performed for 500 ns at 303 K and 1 bar. To analyze the stability of the MD simulation, the root mean square deviation (RMSD), measuring the average deviation of each atom from a reference, was used (71). To analyze the quality of the protein-protein complexes, the interface RMSD (i-RMSD) was used. Furthermore, the quality of a protein-protein docking result was judged by its interface RMSD (i-RMSD). This was fitting for evaluating the docking poses (72), as the i-RMSD was successfully used to differentiate between native and nonnative structures. The i-RMSD is defined as the RMSD of only the atoms within the protein-protein interface (i.e., having an atom from the according touching protein in a 10 Å vicinity). As a third piece of quantitative information, we also visualized the counts of such contacts over the simulation time. These three quantities were evaluated for all MD simulations using custom-written Python scripts based on the Biotite package (73). The protein visualizations were created using PyMol (32).

### Statistical analysis.

The calculated concentrations of the isolated flagella were compared statistically. The repetitive measurements were statistically analyzed using a one-way analysis of variance (ANOVA) with Dunnett’s multiple-comparison test by randomized blocks in GraphPad version 8.2.0 for Windows. The comparisons are based on a statistical significance cutoff of *P ≤ *0.05.

## References

[1] Mondino S, Schmidt S, Rolando M, Escoll P, Gomez-Valero L, Buchrieser C. 2020. Legionnaires’ disease: state of the art knowledge of pathogenesis mechanisms of *Legionella*. Annu Rev Pathol 15:439–466. 10.1146/annurev-pathmechdis-012419-032742. 31657966

[2] Taylor M, Ross K, Bentham R. 2009. *Legionella*, protozoa, and biofilms: interactions within complex microbial systems. Microb Ecol 58:538–547. 10.1007/s00248-009-9514-z. 19365668

[3] Hilbi H, Hoffmann C, Harrison CF. 2011. *Legionella* spp. outdoors: colonization, communication and persistence. Environ Microbiol Rep 3:286–296. 10.1111/j.1758-2229.2011.00247.x. 23761274

[4] Phin N, Parry-Ford F, Harrison T, Stagg HR, Zhang N, Kumar K, Lortholary O, Zumla A, Abubakar I. 2014. Epidemiology and clinical management of Legionnaires’ disease. Lancet Infect Dis 14:1011–1021. 10.1016/S1473-3099(14)70713-3. 24970283

[5] Pagnier I, Merchat M, La Scola B. 2009. Potentially pathogenic amoeba-associated microorganisms in cooling towers and their control. Future Microbiol 4:615–629. 10.2217/fmb.09.25. 19492970

[6] Aurass P, Gerlach T, Becher D, Voigt B, Karste S, Bernhardt J, Riedel K, Hecker M, Flieger A. 2016. Life stage-specific proteomes of *Legionella pneumophila* reveal a highly differential abundance of virulence-associated Dot/Icm effectors. Mol Cell Proteomics 15:177–200. 10.1074/mcp.M115.053579. 26545400 PMC4762515

[7] Swart AL, Harrison CF, Eichinger L, Steinert M, Hilb H. 2018. *Acanthamoeba* and *Dictyostelium* as cellular models for *Legionella* infection. Front Cell Infect Microbiol 8:61. 10.3389/fcimb.2018.00061. 29552544 PMC5840211

[8] Swanson MS, Hammer BK. 2000. *Legionella pneumophila* pathogenesis: a fateful journey from amoebae to macrophages. Annu Rev Microbiol 54:567–613. 10.1146/annurev.micro.54.1.567. 11018138

[9] Jäger J, Marwitz S, Tiefenau J, Rasch J, Shevchuk O, Kugler C, Goldmann T, Steinert M. 2014. Human lung tissue explants reveal novel interactions during *Legionella pneumophila* infections. Infect Immun 82:275–285. 10.1128/IAI.00703-13. 24166955 PMC3911869

[10] Hoppe J, Ünal CM, Thiem S, Grimpe L, Goldmann T, Gaßler N, Richter M, Shevchuk O, Steinert M. 2017. PilY1 promotes *Legionella pneumophila* infection of human lung tissue explants and contributes to bacterial adhesion, host cell invasion, and twitching motility. Front Cell Infect Microbiol 7:63. 10.3389/fcimb.2017.00063. 28326293 PMC5339237

[11] Scheithauer L, Thiem S, Schmelz S, Dellmann A, Büssow K, Brouwer RMHJ, Ünal CM, Blankenfeldt W, Steinert M. 2021. Zinc metalloprotease ProA of *Legionella pneumophila* increases alveolar septal thickness in human lung tissue explants by collagen IV degradation. Cell Microbiol 23:e13313. 10.1111/cmi.13313. 33491325

[12] Cianciotto NP, Eisenstein BI, Mody CH, Toews GB, Engleberg NC. 1989. A *Legionella pneumophila* gene encoding a species-specific surface protein potentiates initiation of intracellular infection. Infect Immun 57:1255–1262. 10.1128/iai.57.4.1255-1262.1989. 2925251 PMC313258

[13] Engleberg NC, Carter C, Weber DR, Cianciotto NP, Eisenstein BJ. 1989. DNA sequence of *mip*, a *Legionella pneumophila* gene associated with macrophage infectivity. Infect Immun 57:1263–1270. 10.1128/iai.57.4.1263-1270.1989. 2925252 PMC313259

[14] Cianciotto NP, Fields BS. 1992. *Legionella pneumophila mip* gene potentiates intracellular infection of protozoa and human macrophages. Proc Natl Acad Sci USA 89:5188–5191. 10.1073/pnas.89.11.5188. 1594630 PMC49255

[15] Fischer G, Bang H, Ludwig B, Mann K, Hacker J. 1992. Mip protein of *Legionella pneumophila* exhibits peptidyl-prolyl-*cis/trans* isomerase (PPlase) activity. Mol Microbiol 6:1375–1383. 10.1111/j.1365-2958.1992.tb00858.x. 1379319

[16] Wintermeyer E, Ludwig B, Steinert M, Schmidt B, Fischer G, Hacker J. 1995. Influence of site specifically altered Mip proteins on intracellular survival of *Legionella pneumophila* in eukaryotic cells. Infect Immun 63:4576–4583. 10.1128/iai.63.12.4576-4583.1995. 7591108 PMC173657

[17] Riboldi-Tunnicliffe A, König B, Jessen S, Weiss MS, Rahfeld J, Hacker J, Fischer G, Hilgenfeld R. 2001. Crystal structure of Mip, a prolylisomerase from Legionella pneumophila. Nat Struct Biol 8:779–783. 10.1038/nsb0901-779. 11524681

[18] Helbig JH, Lück PC, Steinert M, Jacobs E, Witt M. 2001. Immunolocalization of the Mip protein of intracellularly and extracellularly grown *Legionella pneumophila*. Lett Appl Microbiol 32:83–88. 10.1046/j.1472-765x.2001.00861.x. 11169048

[19] Köhler R, Fanghänel J, König B, Lüneberg E, Frosch M, Rahfeld J-U, Hilgenfeld R, Fischer G, Hacker J, Steinert M. 2003. Biochemical and functional analyses of the Mip protein: influence of the N-terminal half and of peptidylprolyl isomerase activity on the virulence of *Legionella pneumophila*. Infect Immun 71:4389–4397. 10.1128/IAI.71.8.4389-4397.2003. 12874317 PMC166037

[20] Galka F, Wai SN, Kusch H, Engelmann S, Hecker M, Schmeck B, Hippenstiel S, Uhlin BE, Steinert M. 2008. Proteomic characterization of the whole secretome of *Legionella pneumophila* and functional analysis of outer membrane vesicles. Infect Immun 76:1825–1836. 10.1128/IAI.01396-07. 18250176 PMC2346698

[21] Schmidt B, Tradler T, Rahfeld JU, Ludwig B, Jain B, Mann K, Rücknagel KP, Janowski B, Schierhorn A, Küllertz G, Hacker J, Fischer G. 1996. A cyclophilin-like peptidyl-prolyl cis/trans isomerase from *Legionella pneumophila*-characterization, molecular cloning and overexpression. Mol Microbiol 21:1147–1160. 10.1046/j.1365-2958.1996.00061.x. 8898384

[22] Fischer G, Tradler T, Zarnt T. 1998. The mode of action of peptidyl prolyl *cis/trans* isomerases in vivo: binding vs. catalysis. FEBS Lett 426:17–20. 10.1016/s0014-5793(98)00242-7. 9598969

[23] Schiene-Fischer C, Aumüller T, Fischer G. 2013. Peptide bond *cis/trans* isomerases: a biocatalysis perspective of conformational dynamics in proteins. Top Curr Chem 328:35–67. 10.1007/128_2011_151. 21598101

[24] Ceymann A, Horstmann M, Ehses P, Schweimer K, Paschke A-K, Steinert M, Faber C. 2008. Solution structure of the *Legionella pneumophila* Mip-rapamycin complex. BMC Struct Biol 8:17. 10.1186/1472-6807-8-17. 18366641 PMC2311308

[25] Horstmann M, Ehses P, Schweimer K, Steinert M, Kamphausen T, Fischer G, Hacker J, Rösch P, Faber C. 2006. Domain motions of the Mip protein from Legionella pneumophila. Biochemistry 45:12303–12311. 10.1021/bi060818i. 17014083

[26] Wagner C, Khan AS, Kamphausen T, Schmausser B, Unal C, Lorenz U, Fischer G, Hacker J, Steinert M. 2007. Collagen binding protein Mip enables *Legionella pneumophila* to transmigrate through a barrier of NCI-H292 lung epithelial cells and extracellular matrix. Cell Microbiol 9:450–462. 10.1111/j.1462-5822.2006.00802.x. 16953800

[27] Söderberg MA, Cianciotto NP. 2008. A *Legionella pneumophila* peptidyl-prolyl *cis-trans* isomerase present in culture supernatants is necessary for optimal growth at low temperatures. Appl Environ Microbiol 74:1634–1638. 10.1128/AEM.02512-07. 18165359 PMC2258609

[28] Ünal C, Schwedhelm KF, Thiele A, Weiwad M, Schweimer K, Frese F, Fischer G, Hacker J, Faber C, Steinert M. 2011. Collagen IV-derived peptide binds hydrophobic cavity of *Legionella pneumophila* Mip and interferes with bacterial epithelial transmigration. Cell Microbiol 13:1558–1572. 10.1111/j.1462-5822.2011.01641.x. 21794054

[29] Teufel F, Almagro Armenteros JJ, Johansen AR, Gíslason MH, Pihl SI, Tsirigos KD, Winther O, Brunak S, von Heijne G, Nielsen H. 2022. SignalP 6.0 predicts all five types of signal peptides using protein language models. Nat Biotechnol 40:1023–1025. 10.1038/s41587-021-01156-3. 34980915 PMC9287161

[30] Appelt S, Heuner K. 2017. The flagellar regulon of Legionella - a review. Front Cell Infect Microbiol 7:454. 10.3389/fcimb.2017.00454. 29104863 PMC5655016

[31] Bolon DN, Grant RA, Baker TA, Sauer RT. 2004. Nucleotide-dependent substrate handoff from the SspB adaptor to the AAA+ ClpXP protease. Mol Cell 16:343–350. 10.1016/j.molcel.2004.10.001. 15525508

[32] Schrödinger L, DeLano W. 2020. The PyMol Molecular Graphics System, Version 2.0 Schrödinger LLC, available at: http://www.pymol.org/pymol.

[33] Leaver-Fay A, Tyka M, Lewis SM, Lange OF, Thompson J, Jacak R, Kaufman K, Renfrew PD, Smith CA, Sheffler W, Davis IW, Cooper S, Treuille A, Mandell DJ, Richter F, Ban Y-EA, Fleishman SJ, Corn JE, Kim DE, Lyskov S, Berrondo M, Mentzer S, Popović Z, Havranek JJ, Karanicolas J, Das R, Meiler J, Kortemme T, Gray JJ, Kuhlman B, Baker D, Bradley P. 2011. Rosetta3: an object-oriented software suite for the simulation and design of macromolecules. Methods Enzymol 487:545–574. 10.1016/B978-0-12-381270-4.00019-6. 21187238 PMC4083816

[34] Wang F, Burrage AM, Postel S, Clark RE, Orlova A, Sundberg EJ, Kearns DB, Egelman EH. 2017. A structural model of flagellar filament switching across multiple bacterial species. Nat Commun 8:960. 10.1038/s41467-017-01075-5. 29038601 PMC5643327

[35] Dietrich C, Heuner K, Brand B, Hacker J, Steinert M. 2001. Flagellum of *Legionella pneumophila* positively affects the early phase of infection of eukaryotic host cells. Infect Immun 69:2116–2122. 10.1128/IAI.69.4.2116-2122.2001. 11254565 PMC98137

[36] Cahoon LA, Freitag NE. 2015. Identification of conserved and species-specific functions of the *Listeria monocytogenes* PrsA2 secretion chaperone. Infect Immun 83:4028–4041. 10.1128/IAI.00504-15. 26216425 PMC4567654

[37] Wiemels RE, Cech SM, Meyer NM, Burke CA, Weiss A, Parks AR, Shaw LN, Carroll RK. 2017. An intracellular peptidyl-prolyl cis/trans isomerase is required for folding and activity of the *Staphylococcus aureus* secreted virulence factor nuclease. J Bacteriol 199:e00453-16. 10.1128/JB.00453-16. 27795319 PMC5165095

[38] Stewart DE, Sarkar A, Wampler JE. 1990. Occurrence and role of cis peptide bonds in protein structures. J Mol Biol 214:253–260. 10.1016/0022-2836(90)90159-J. 2370664

[39] Ünal CM, Steinert M. 2014. Microbial peptidyl-prolyl cis/trans isomerases (PPIases): virulence factors and potential alternative drug targets. Microbiol Mol Biol Rev 78:544–571. 10.1128/MMBR.00015-14. 25184565 PMC4187684

[40] Ünal CM, Karagöz MS, Berge M, Priebe C, Borrero De Acuna JM, Wissing J, Jänsch L, Jahn D, Steinert M. 2019. Pleiotropic *Clostridioides difficile* cyclophilin PpiB controls cysteine-tolerance, toxin production, the central metabolism and multiple stress responses. Front Pharmacol 10:340. 10.3389/fphar.2019.00340. 31024308 PMC6459899

[41] Bzdyl NM, Scott NE, Norville IH, Scott AE, Atkins T, Pang S, Sarovich DS, Coombs G, Inglis TJJ, Kahler CM, Sarkar-Tyson M. 2019. Peptidyl-prolyl isomerase *ppiB* is essential for proteome homeostasis and virulence in *Burkholderia pseudomallei*. Infect Immun 87:e00528-19. 10.1128/IAI.00528-19. 31331957 PMC6759293

[42] Ünal CM, Berges M, Smit N, Schiene-Fischer C, Priebe C, Strowig T, Jahn D, Steinert M. 2018. PrsA2 (CD630_35000) of Clostridioides difficile is an active parvulin-type PPIase and a virulence modulator. Front Microbiol 9:2913. 10.3389/fmicb.2018.02913. 30564207 PMC6288519

[43] Debroy S, Aragon V, Kurtz S, Cianciotto NP. 2006. *Legionella pneumophila* Mip, a surface-exposed peptidylproline cis-trans-isomerase, promotes the presence of phospholipase C-like activity in culture supernatants. Infect Immun 74:5152–5160. 10.1128/IAI.00484-06. 16926407 PMC1594821

[44] Rasch J, Ünal CM, Klages A, Karsli Ü, Heinsohn N, Brouwer RMHJ, Richter M, Dellmann A, Steinert M. 2019. PPIases Mip and PpiB of Legionella pneumophila contribute to surface translocation, growth at suboptimal temperature and infection. Infect Immun 15 pii:IAI 00939-17. 10.1128/IAI.00939-17 PMC6300627 30323027

[45] Rasch J, Krüger S, Fontvieille D, Ünal CM, Michel R, Labrosse A, Steinert M. 2016. Legionella-protozoa-nematode interactions in aquatic biofilms and influence of Mip on Caenorhabditis elegans colonization. Int J Med Microbiol 306:443–451. 10.1016/j.ijmm.2016.05.012. 27288243

[46] Scheuplein NJ, Bzdyl NM, Kibble EA, Lohr T, Holzgrabe U, Sarkar-Tyson M. 2020. Targeting protein folding: a novel approach for the treatment of pathogenic bacteria. J Med Chem 63:13355–13388. 10.1021/acs.jmedchem.0c00911. 32786507

[47] Rasch J, Theuerkorn M, Ünal C, Heinsohn N, Tran S, Fischer G, Weiwad M, Steinert M. 2015. Novel cycloheximide derivatives targeting the moonlighting protein Mip exhibit specific antimicrobial activity against Legionella pneumophila. Front Bioeng Biotechnol 3(:41. 10.3389/fbioe.2015.00041. 25870856 PMC4376002

[48] Rasch J, Ünal C, Steinert M. 2014. Peptidylprolyl *cis-trans* isomerases of *Legionella pneumophila*: virulence, moonlighting and novel therapeutic targets. Biochem Soc Trans 42:1728–1733. 10.1042/BST20140202. 25399597

[49] Juli C, Sippel M, Jäger J, Thiele A, Weiwad M, Schweimer K, Rösch P, Steinert M, Sotriffer CA, Holzgrabe U. 2011. Pipecolic acid derivatives as small-molecule inhibitors of the Legionella MIP protein. J Med Chem 54:277–283. 10.1021/jm101156y. 21142106

[50] Pomplun S, Sippel C, Hähle A, Tay D, Shima K, Klages A, Ünal CM, Rieß B, Toh HT, Hansen G, Yoon HS, Bracher A, Preiser PR, Rupp J, Steinert M, Hausch F. 2018. Chemogenomic profiling of human and microbial FK506-binding proteins. J Med Chem 61:3660–3673. 10.1021/acs.jmedchem.8b00137. 29578710

[51] Levchenko I, Seidel M, Sauer RT, Baker TA. 2000. A specificity-enhancing factor for the ClpXP degradation machine. Science 289:2354–2356. 10.1126/science.289.5488.2354. 11009422

[52] Yin J, Ding M, Zha F, Zhang J, Meng Q, Yu Z. 2021. Stringent starvation protein regulates prodiginine biosynthesis via affecting siderophore production in *Pseudoalteromonas* sp. strain R3. Appl Environ Microbiol 87:e02949-20. 10.1128/AEM.02949-20. 33483309 PMC8091607

[53] Scherer CA, Cooper E, Miller SI. 2000. The *Salmonella* type III secretion translocon protein SspC is inserted into the epithelial cell plasma membrane upon infection. Mol Microbiol 37:1133–1145. 10.1046/j.1365-2958.2000.02066.x. 10972831

[54] Tomoyasu T, Ohkishi T, Ukyo Y, Tokumitsu A, Takaya A, Suzuki M, Sekiya K, Matsui H, Kutsukake K, Yamamoto T. 2002. The ClpXP ATP-dependent protease regulates flagellum synthesis in *Salmonella enterica* serovar Typhimurium. J Bacteriol 184:645–653. 10.1128/JB.184.3.645-653.2002. 11790733 PMC139528

[55] Molofsky AB, Shetron-Rama LM, Swanson MS. 2005. Comonents of the *Legionella pneumophila* flagellar regulon contribute to multiple virulence traits, including lysosome avoidance and macrophage death. Infect Immun 73:5720–5734. 10.1128/IAI.73.9.5720-5734.2005. 16113289 PMC1231111

[56] Hammer BK, Tateda ES, Swanson MS. 2002. A two-component regulator induces the transmission phenotype of stationary-phase *Legionella pneumophila*. Mol Microbiol 44:107–118. 10.1046/j.1365-2958.2002.02884.x. 11967072 PMC13220096

[57] Heuner K, Steinert M. 2003. The flagellum of *Legionella pneumophila* and its link to the expression of the virulent phenotype. Int J Med Microbiol 293:133–143. 10.1078/1438-4221-00259. 12868650

[58] Forstneric V, Ivicak-Kocjan K, Plaper T, Jerala R, Bencina M. 2017. The role of the C-terminal D0 domain of flagellin in activation of Toll like receptor 5. PLoS Pathog 13:e1006574. 10.1371/journal.ppat.1006574. 28827825 PMC5578693

[59] Song WS, Jeon JY, Namgung B, Hong M, Yoon S. 2017. A conserved TLR5 binding and activation hot spot on flagellin. Scient Rep 7:40878. 10.1038/srep40878 PMC5247705 28106112

[60] Borrero-de Acuña JM, Rohde M, Wissing J, Jänsch L, Schobert M, Molinari G, Timmis KN, Jahn M, Jahn D. 2016. Protein network of the *Pseudomonas aeruginosa* denitrification apparatus. J Bacteriol 198:1401–1413. 10.1128/JB.00055-16. 26903416 PMC4836231

[61] Hanahan D. 1983. Studies on transformation of *Escherichia coli* with plasmids. J Mol Biol 166:557–580. 10.1016/s0022-2836(83)80284-8. 6345791

[62] Montie TC, Craven RC, Holder IA. 1982. Flagellar preparations from *Pseudomonas aeruginosa*: isolation and characterization. Infect Immun 35:281–288. 10.1128/iai.35.1.281-288.1982. 6797949 PMC351027

[63] Helbig JH, Ludwig B, Lück PC, Groh A, Witzleb W, Hacker J. 1995. Monoclonal antibodies to *Legionella* Mip proteins recognize genus- and species-specific epitopes. Clin Diagn Lab Immunol 2:160–165. 10.1128/cdli.2.2.160-165.1995. 7535177 PMC170120

[64] Neuhoff V, Arold N, Taube D, Ehrhardt W. 1988. Improved staining of proteins in polyacrylamide gels including isoelectric focusing gels with clear background at nanogram sensitivity using Coomassie Brilliant Blue G-250 and R-250. Electrophoresis 9:255–262. 10.1002/elps.1150090603. 2466658

[65] Blum H, Beier H, Gross HJ. 1987. Improved silver staining of plant proteins RNA and ist in polyacrylamide gels. Electrophoresis 8:93–99. 10.1002/elps.1150080203.

[66] Cianciotto NP, Bangsborg JM, Eisenstein BI, Engleberg NC. 1990. Identification of *mip*-like genes in the genus *Legionella*. Infect Immun 58:2912–2918. 10.1128/iai.58.9.2912-2918.1990. 2387627 PMC313586

[67] Heuner K, Bender-Beck L, Brand BC, Lück PC, Mann KH, Marre R, Ott M, Hacker J. 1995. Cloning and genetic characterization of the flagellum subunit gene (flaA) of *Legionella pneumophila* serogroup 1. Infect Immun 63:2499–2507. 10.1128/iai.63.7.2499-2507.1995. 7790062 PMC173334

[68] Jumper J, Evans R, Pritzel A, Green T, Figurnov M, Ronneberger O, Tunyasuvunakool K, Bates R, Žídek A, Potapenko A, Bridgland A, Meyer C, Kohl SAA, Ballard AJ, Cowie A, Romera-Paredes B, Nikolov S, Jain R, Adler J, Back T, Petersen S, Reiman D, Clancy E, Zielinski M, Steinegger M, Pacholska M, Berghammer T, Bodenstein S, Silver D, Vinyals O, Senior AW, Kavukcuoglu K, Kohli P, Hassabis D. 2021. Highly accurate protein structure prediction with AlphaFold. Nature 596:583–589. 10.1038/s41586-021-03819-2. 34265844 PMC8371605

[69] Abraham MJ, Murtola T, Schulz R, Páll S, Smith JC Hess B, Lindahl E. 2015. GROMACS: high performance molecular simulations through multi-level parallelism from laptops to supercomputers. SoftwareX 1–2:19–25. 10.1016/j.softx.2015.06.001.

[70] Vanommeslaeghe K, Hatcher E, Acharya C, Kundu S, Zhong S, Shim J, Darian E, Guvench O, Lopes P, Vorobyov I, Mackerell AD. 2010. CHARMM general force field: a force field for drug-like molecules compatible with the CHARMM all-atom additive biological force fields. J Comput Chem 31:671–690. 10.1002/jcc.21367. 19575467 PMC2888302

[71] Benson NC, Daggett VA. 2012. Comparison of multiscale methods for the analysis of molecular dynamics simulations. J Phys Chem B 116:8722–8731. 10.1021/jp302103t. 22494262 PMC3406285

[72] Jandova Z, Vargiu AV, Bonvin AMJJ. 2021. Native or non-native protein-protein docking models? Molecular dynamics to the rescue. J Chem Theory Comput 17:5944–5954. 10.1021/acs.jctc.1c00336. 34342983 PMC8444332

[73] Kunzmann P, Hamacher K. 2018. Biotite: a unifying open source computational biology framework in Python. BMC Bioinformatics 19:346. 10.1186/s12859-018-2367-z. 30285630 PMC6167853

[74] Jepras RI, Fitzgeorge RB, Baskerville A. 1985. A comparison of virulence of two strains of *Legionella pneumophila* based on experimental aerosol infection of guinea-pigs. J Hyg (Lond) 95:29–38. 10.1017/s0022172400062252. 4020112 PMC2129515

