# Supplementary material for: Legionella pneumophila PPIase Mip Interacts with the Bacterial Proteins SspB, Lpc2061, and FlaA and Promotes Flagellation
Source: Infect Immun. 2022 Oct 31;90(11):e00276-22. doi: 10.1128/iai.00276-22 (PMC9670971; doi:10.1128/iai.00276-22)
Supplement: Supplemental file 10 — Fig. S1 to S8 and Tables S1 to S4. Download iai.00276-22-s0001.pdf, PDF file, 1.7 MB [file iai.00276-22-s0001.pdf]

SUPPLEMENTAL FIGURES

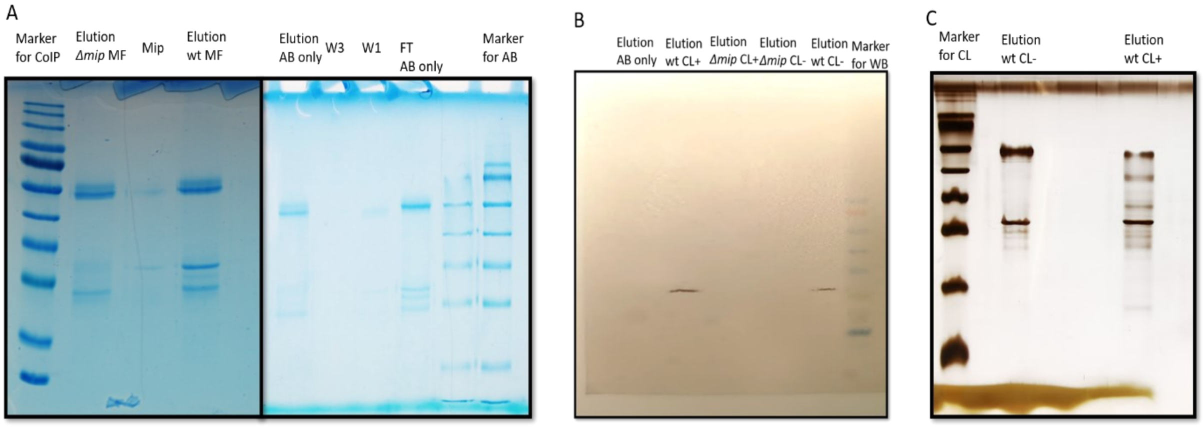

FIG S1. Immuno-detection of Mip in eluates derived from bacterial membrane fractions (MF). Uncropped images of Fig. 1 show (A) Coomassie stained SDS gels, (B) the corresponding Western blot, and (C) the silver-stained gel with and without crosslinking (CL) after co-immunoprecipitation (CoIP) and reveal bands which refer to purified Mip. Moreover, elution fractions show heavy and light chains from antibodies (AB) at 50 and 25 kDa in Coomassie stains. FT, flow through; W, wash.

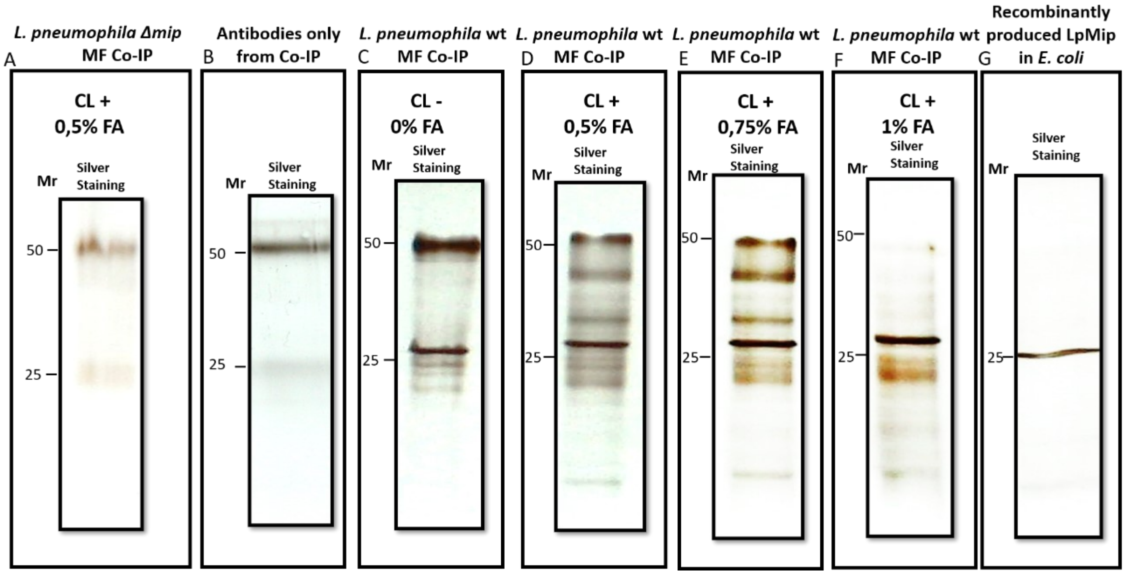

FIG S2. Identification of bacterial interaction partners of Mip by co-immunoprecipitation. Bacterial cell membrane fractions (MF) were solubilized and co-immunoprecipitated (Co-IP) with a mixture of monoclonal Mip-antibodies 2D8 and 22/1. Eluted proteins were resolved by SDS-PAGE and silver stained. Purification of Mip interaction partners was optimized by adding different concentrations of crosslinking (CL) agent formaldehyde (FA) to the bacterial cultures prior harvest.

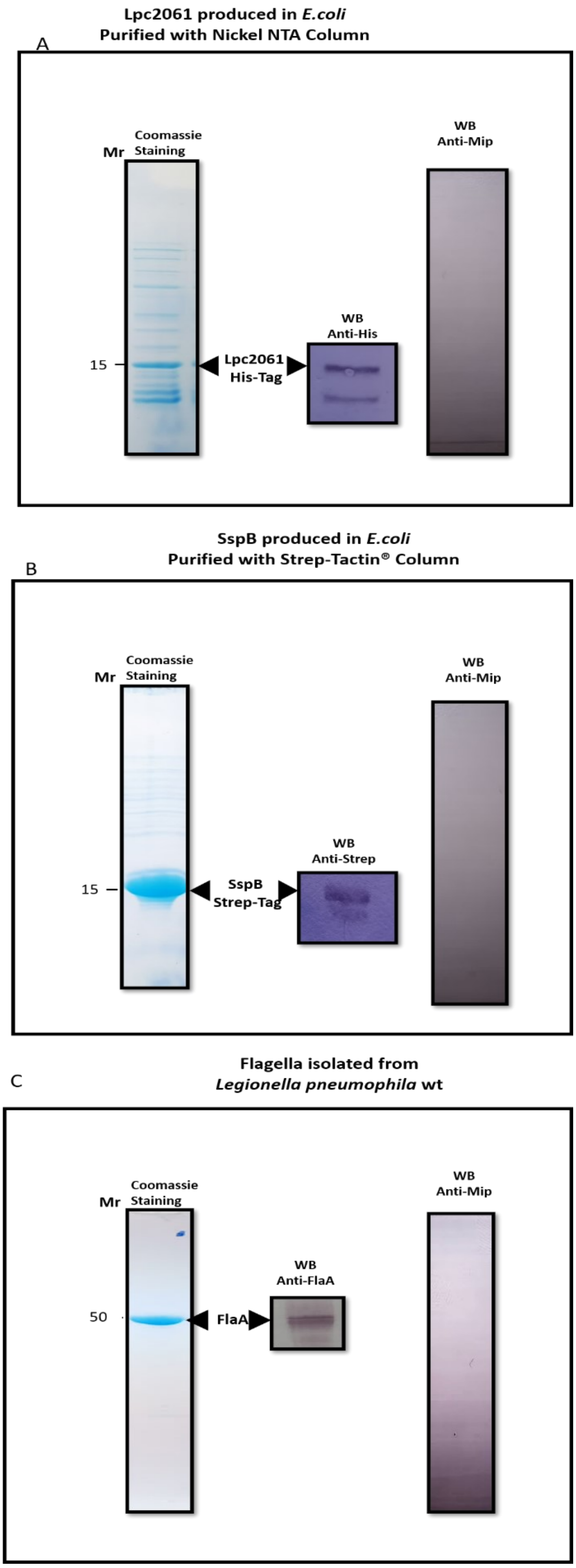

FIG S3. Anti-Mip antibodies 2D8 and 22/1 which were used in the co-immunoprecipitation showed no binding to purified interaction partners as shown by Western blotting (WB).

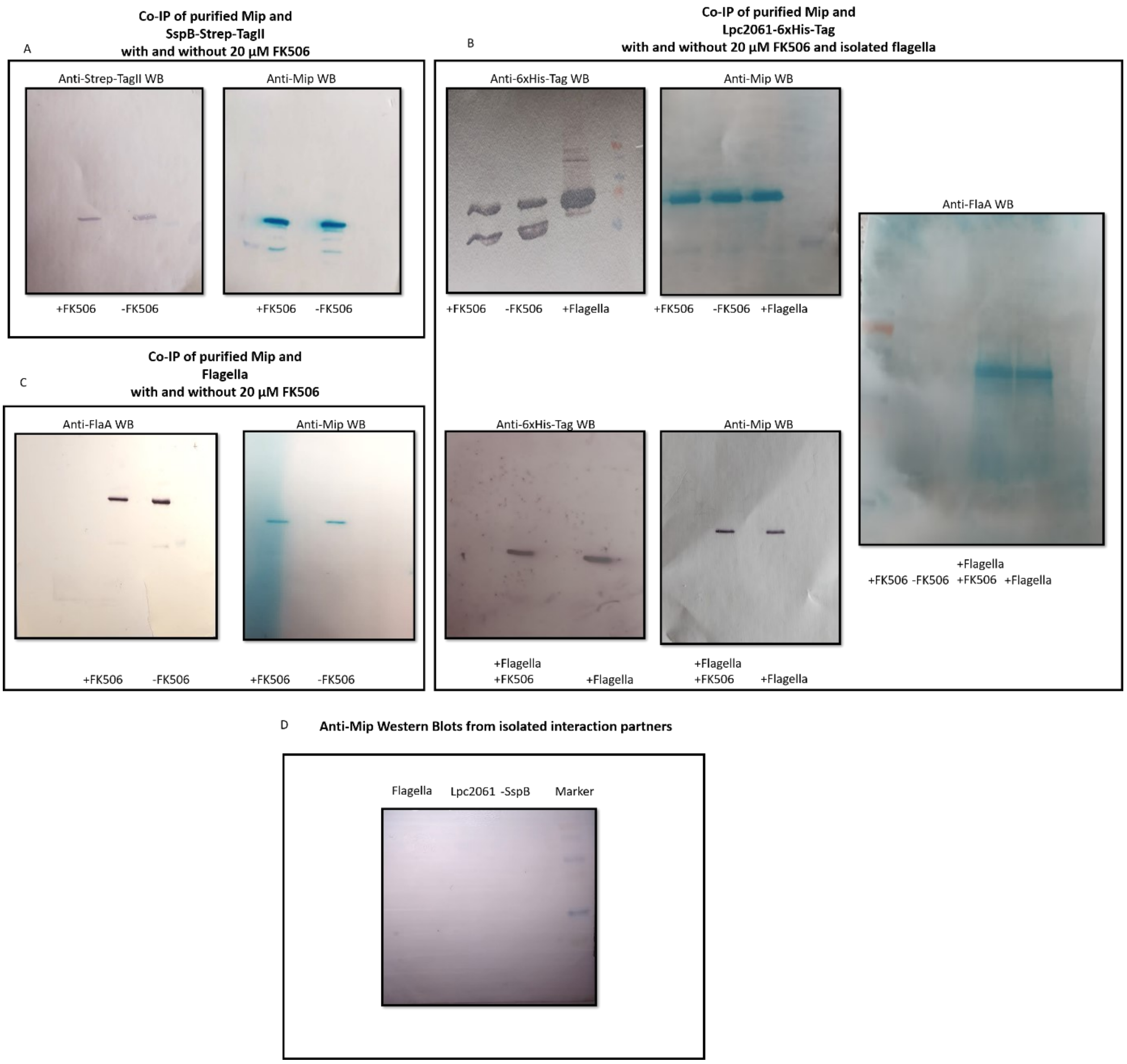

FIG S4. Validation of Mip interaction partners by co-immunoprecipitation (Co-IP) of SspB-Strep-TagII, Lpc2061-6xHis-Tag, and purified native flagellin of *L. pneumophila*. Recombinant Mip was immobilized on magnetic DynaBeads coated with Protein G, 2D8 and 22/1 antibodies and loaded with 1 mg of each purified interaction partner. All co-eluates were resolved by SDS-PAGE. The associations of the interaction partners were confirmed by Western blotting (WB).

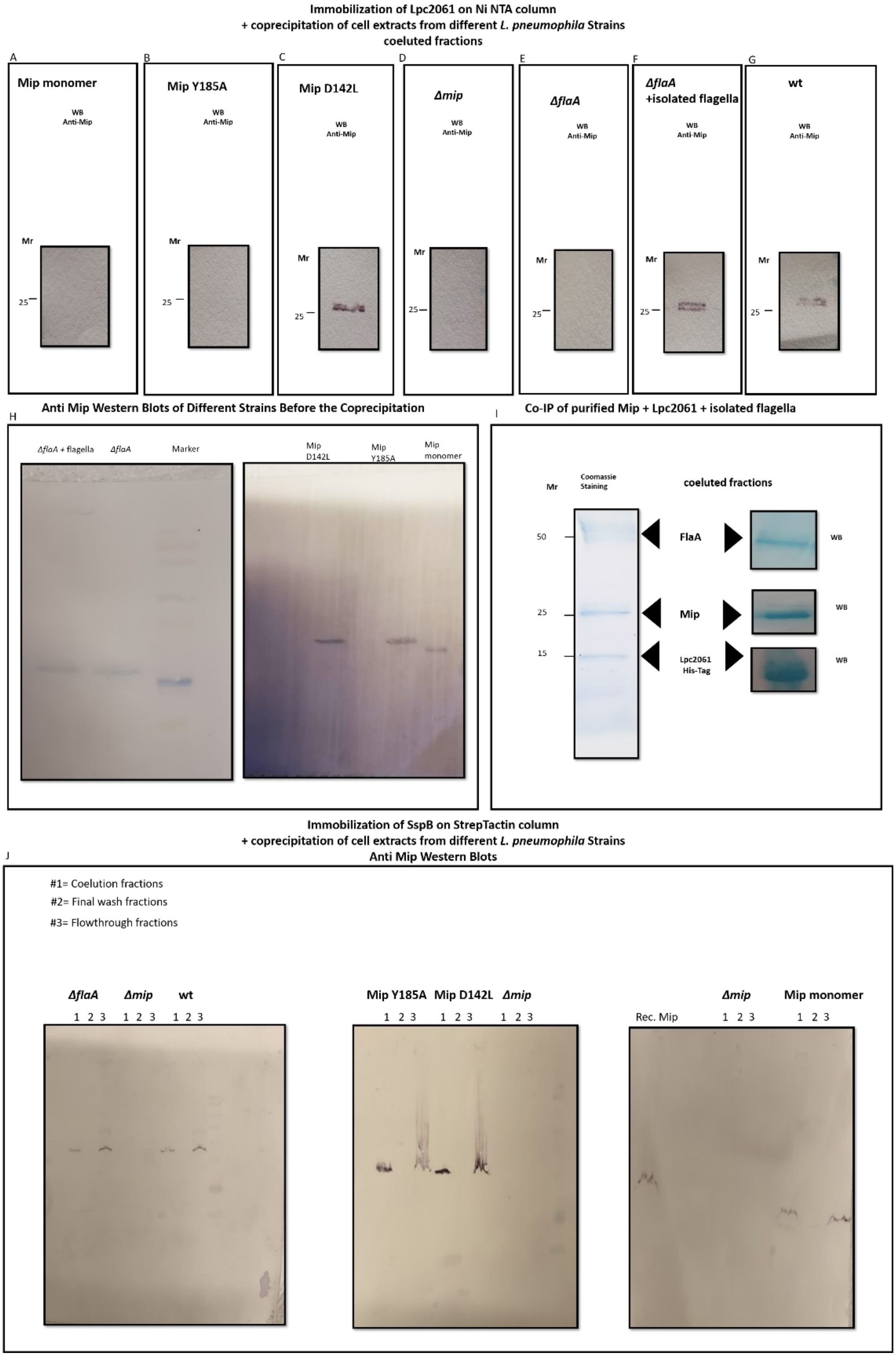

FIG S5. Cell extracts of *L. pneumophila* strains which produce N-terminally truncated Mip monomers, Mip variants with single amino acid substitutions, or cell extracts of *mip*-and *flaA*-negative strains were analyzed for their ability to co-precipitate with Lpc2061 on Nickel-NTA and SspB on StrepTactin columns. (A-H) Co-precipitation of Mip variants and Lpc2061 was analyzed by Western blotting. Uncropped images of Western blots before co-precipitation show equal amounts of Mip in *L. pneumophila* strains lacking FlaA or expressing different Mip variants. (I) CoIP of purified Lpc2061 and isolated flagella with recombinant Mip. (J) Co-precipitation of Mip variants and SspB was analyzed by Western blotting. Western blots show the Mip-SspB binding in the co-elution fractions (#1), final wash fractions show the purity of the column before the co-elution (#2) and flowthrough fractions show the Mip presence before co-precipitations (#3). Immobilisation of SspB resulted in binding of the different Mip variants. The SspB-Mip binding was not dependent on FlaA.

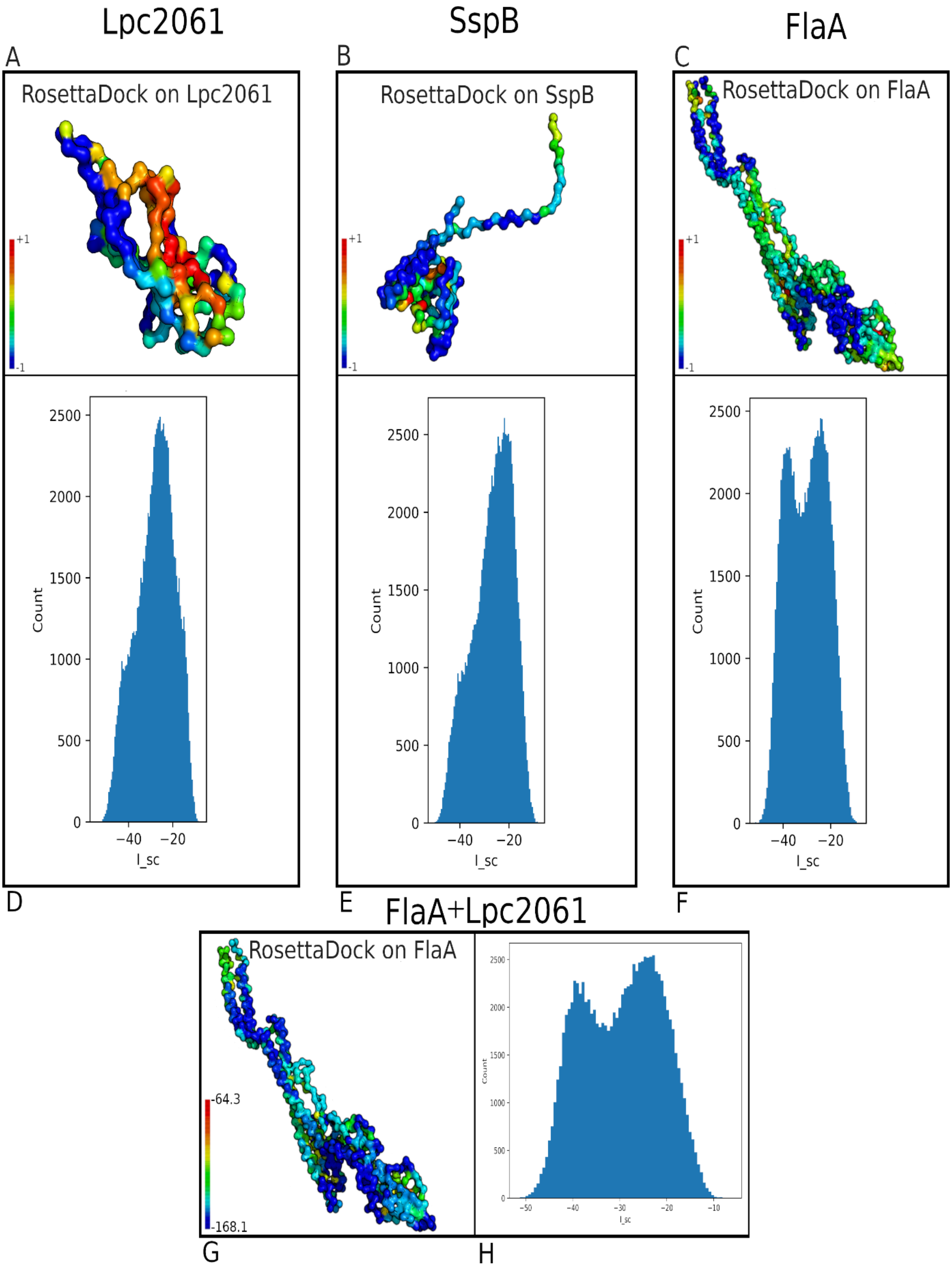

FIG S6. Global docking and interface score histograms of the Mip interaction partners Lpc2061, SspB and FlaA. The best poses with contact to the protein of the entire ensemble are depicted in the plots.

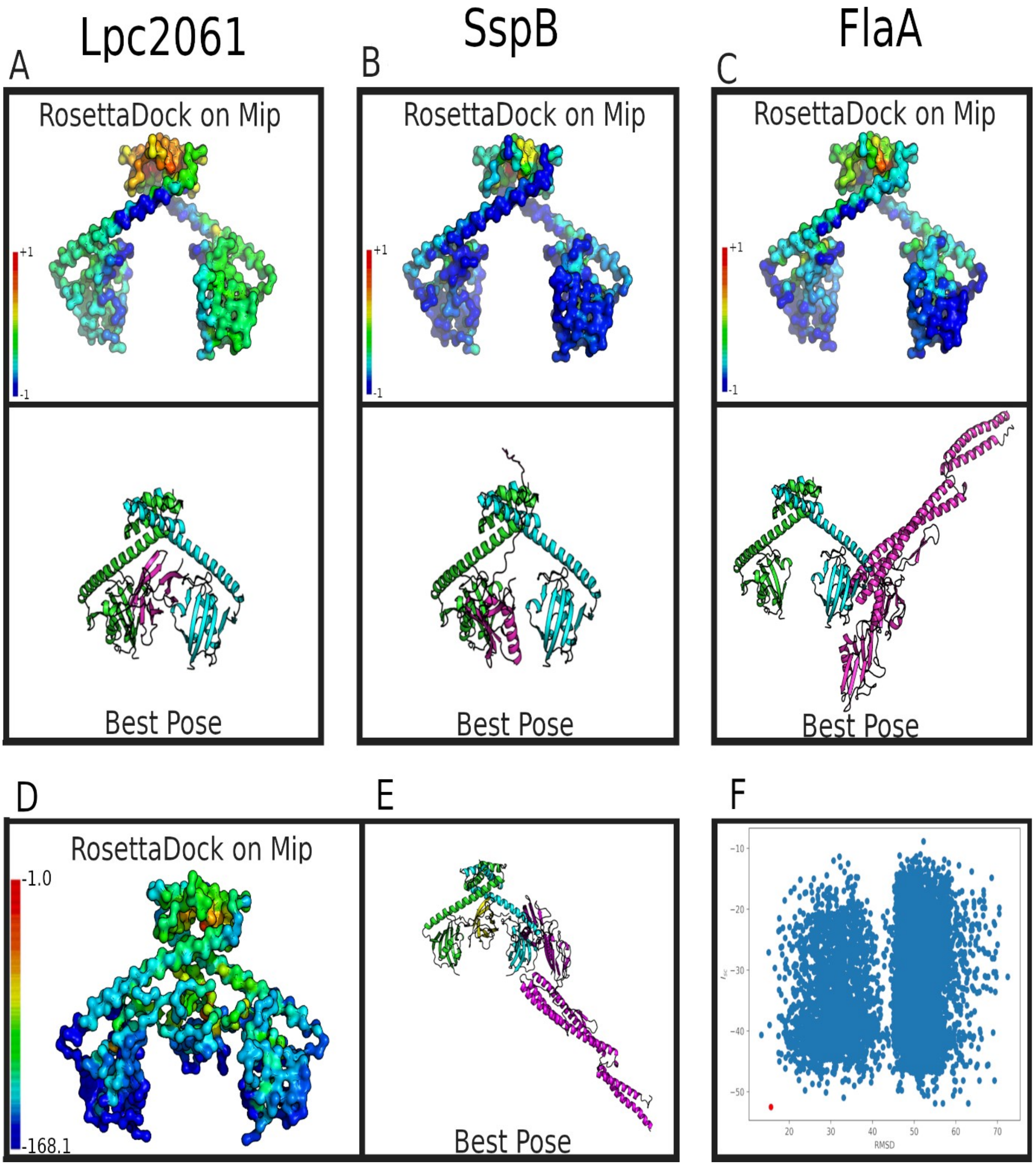

FIG S7. Global docking of interaction partners to Mip using RosettaDock and scatter plot of the minimum RMSD between each pose from the flagellar filament docking and the MD trajectory for Mip with Lpc2061 and FlaA.The upper row shows the back views (rotated by 180 degrees around the z axis) of the docking results presented in Figure 6. The second row shows the best docking pose for comparison with the color-coded interface score on the protein surface (interaction partner in magenta). The lower row shows the docking of FlaA to Mip + Lpc2061, the best pose and the scatter plot of the minimum RMSD between each pose from the flagellar filament docking and the MD trajectory for Mip with Lpc2061 + FlaA. The red marked point in the lower left corner represents the best pose of the filament docking.

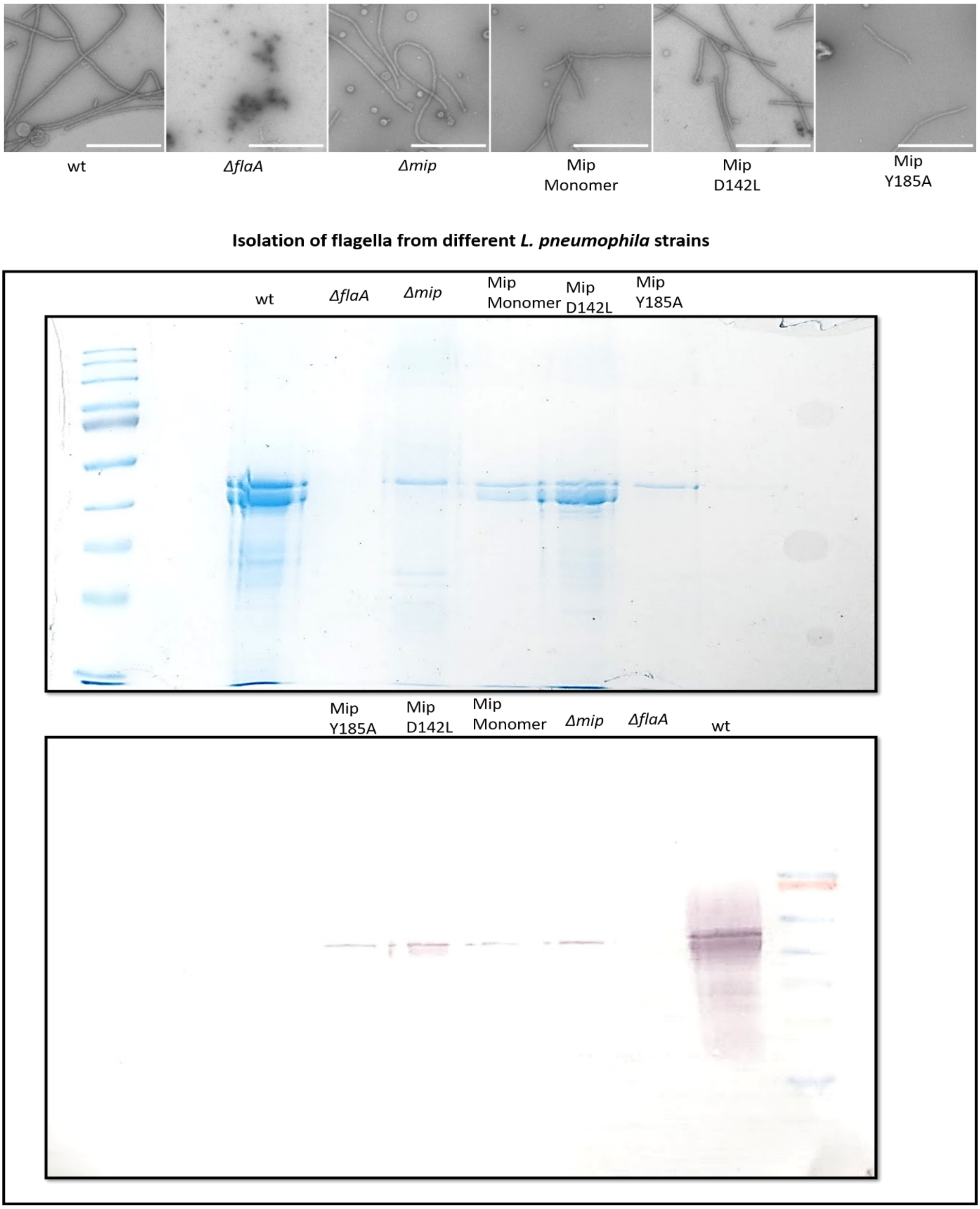

FIG S8. Electron micrographs of isolated flagella from *L. pneumophila* wildtype and mutant strains. Upper panels scale bar 500 nm. Flagella isolated from 10^{14} bacteria of the *L. pneumophila* wild type (wt) strain or the isogenic Δ*mip* mutant were separated by SDS-PAGE and Coomassie stained. The identity of FlaA was confirmed by Western blotting.

SUPPLEMENTAL TABLES

TABLE S1 | Quantification of protein bands and concentrations from isolated flagella with same number of bacteria

**Flagella isolation from different *Legionella* strains**	**Intensities of the whole lane[a]**			**Band intensity at FlaA size**			**Concentration of isolated flagella in mg/mL**		
Repeat	1st	2nd	3rd	1st	2nd	3rd	1st	2nd	3rd
wt	15845	16454	16694	13970	14879	15012	5,2	4,1	6,56
Δ*flaA*	4288	4503	4838	3828	4013	4253	0,78	0,67	0,83
Δ*mip*	5709	6619	6191	5632	6344	5786	1,1	0,89	1,02
Mip Monomer	4956	5553	5564	4502	4985	4879	1,2	1,32	1,23
Mip D142L	11234	9289	9056	9692	8434	7800	3,95	2,33	4,62
Mip Y185A	4764	4945	5094	3984	4356	4415	0,96	0,85	0,89

[a]The intensities of Coomassie staining from flagella isolation of different *L. pneumophila* strains. The numbers are areas of the plots from the bands of isolated flagella and the band intensities at ~50kDa created with imageJ.

TABLE S2 | ANOVA from quantified concentrations

**Dunnett's multiple comparisons test**					
**Concentration in µg/ml**	**Mean Diff,**	**95,00% CI of diff,**	**Significant?**	**Summary[a]**	**Adjusted P Value**
wt vs. Δ*flaA*	4527	2870 to 6183	Yes	****	<0,0001
wt vs. Δ*mip*	4283	2627 to 5940	Yes	****	<0,0001
wt vs. mono	4037	2380 to 5693	Yes	****	<0,0001
wt vs. D142L	1657	-0,06199 to 3313	No	Ns	0,05
wt vs. Y185A	4387	2730 to 6043	Yes	****	<0,0001

[a]The measurements of concentrations are statistically analysed via using GraphPad, one-way ANOVA and the Dunnett's test is taken for the calculation of significancy compared to the wt strains. * is p< 0,05 **shows p<0,01 *** depicts p<0,001 ns stands for not significant

TABLE S3 | Quantification of concentrations from isolated flagella with same number of bacteria

**Flagella isolation from different *Legionella* strains**	**Concentration of isolated flagella in mg/mL**		
Repeat	1st	2nd	3rd
Wt	6746	7182	8972
Δ*flaA*	2383	2012	1721
wt +20μM FK506	3748	2578	2497

TABLE S4 | ANOVA from quantified concentrations

Dunnett's multiple comparisons test

**Concentration in µg/ml**	**Mean Diff,**	**95,00% CI of diff,**	**Significant?**	**Summary[a]**	**Adjusted P Value**
wt vs. flaA	5595	3626 to 7563	Yes	***	0,0003
wt vs. wt + FK506	4692	2724 to 6661	Yes	***	0,0008

[a]The measurements of concentrations are statistically analysed via using GraphPad, one-way ANOVA and the Dunnett´s multiple comparisons test is taken for the calculation of significancy compared to the wt strains. * is p< 0,05 **shows p<0,01 *** depicts p<0,001 ns stands for not significant
